# Proteomic Analysis Reveals a Mitochondrial Remodeling of βTC3 Cells in Response to Nanotopography

**DOI:** 10.3389/fcell.2020.00508

**Published:** 2020-07-29

**Authors:** Elisa Maffioli, Alessandra Galli, Simona Nonnis, Algerta Marku, Armando Negri, Claudio Piazzoni, Paolo Milani, Cristina Lenardi, Carla Perego, Gabriella Tedeschi

**Affiliations:** ^1^Department of Veterinary Medicine, University of Milano, Milan, Italy; ^2^Centre for Nanostructured Materials and Interfaces, University of Milano, Milan, Italy; ^3^Department of Pharmacological and Biomolecular Sciences, University of Milano, Milan, Italy; ^4^Department of Physics, University of Milano, Milan, Italy

**Keywords:** insulin-secreting cells, mitochondria, mechanotransduction, proteomics, nanosubstrates

## Abstract

Recently, using cluster-assembled zirconia substrates with tailored roughness produced by supersonic cluster beam deposition, we demonstrated that β cells can sense nanoscale features of the substrate and can translate these stimuli into a mechanotransductive pathway capable of preserveing β-cell differentiation and function *in vitro* in long-term cultures of human islets. Using the same proteomic approach, we now focused on the mitochondrial fraction of βTC3 cells grown on the same zirconia substrates and characterized the morphological and proteomic modifications induced by the nanostructure. The results suggest that, in βTC3 cells, mitochondria are perturbed by the nanotopography and activate a program involving metabolism modification and modulation of their interplay with other organelles. Data were confirmed in INS1E, a different β-cell model. The change induced by the nanostructure can be pro-survival and prime mitochondria for a metabolic switch to match the new cell needs.

## Introduction

Cells are competent at precisely sensing the physical condition (rigidity and nanoscale topography) of their microenvironment, and the perceived information has a strong impact on the cell signaling, behavior, and fate. This phenomenon, i.e., the conversion of microenvironmental biophysical information into corresponding cellular responses, is defined as mechanotransduction: the extracellular matrix (ECM) configuration affects the assembly of integrin adhesion complexes (IAC) and their signaling. This impacts the nuclear architecture and mechanosensitive transcription factors, eventually leading to modifications of the cell program (Chen et al., 2014; Schulte et al., 2016; Galli et al., 2018, 2020; Maffioli et al., 2018; Chighizola et al., 2019).

The ECM is made by a mixture of proteins whose specific organization and composition differ extensively across the tissues and determine the matrix rigidity, structure, and topographical configuration (Hynes, 2009; Gasiorowski et al., 2013).

This versatility adds an extra level of intricacy in the complex interplay between cells and ECM. To gain a basic understanding of how cells process topographical surface information, reductionist approaches, such as artificial microenvironments with simplified regular topographical cues obtained by top-down micro- and nanofabrication techniques, have been used. As an alternative, special tools available include nanostructured surfaces resulting from the assembling of nanoparticles deposited by Supersonic Cluster Beam Deposition (SCBD; Schulte et al., 2017). Cluster-assembled surfaces are characterized by random nanoscale roughness that can be carefully controlled and can range from a few nanometers up to some tens of nanometers, allowing precise engineering of surface features at the nanoscale, reproducing thereby *in vivo* ECM structural characteristics (Wegner et al., 2006; Gasiorowski et al., 2013; Podestà et al., 2015; Schulte et al., 2016, 2017).

Recently, using cluster-assembled zirconia substrates with tailored roughness produced by SCBD, we provide evidence that β-cells can perceive the nanotopography of the substrate and can convert this information into a mechanotransductive process, which preserves β-cell differentiation and function in long-term *in vitro* culture of human islets (Galli et al., 2018, 2020).

We characterized through use of proteomics the molecular mechanisms involved gaining new information on the ability of islets to transduce the topographical information in a biological program. Indeed, we demonstrated that human β cells, using integrines as mechanosensors, can channel mechanical forces, through actin fibers, directly to the nucleus where they cause nuclear envelope modifications and activation of a specific transcriptional program which supports β-cell survival and preserves β-cell differentiation and insulin secretion. This phenomenon depends on the nanoscale surface topography since only cells grown on cluster-assembled films maintain the functional phenotype (Galli et al., 2018, 2020). Whether other organelles are involved in the phenomenon is unknown.

β-cell survival and function closely rely on mitochondria activity. As in the other cells, mitochondria provide the energy necessary for cell survival, control several biosynthetic processes, modulate Ca^2+^ signaling, and integrate different apoptotic stimuli (Maechler and Wollheim, 2001). In β cells, mitochondria also couple nutrient metabolism to electrical activity, ensuring appropriate insulin secretion. The model of β-cell stimulus-secretion coupling holds that after its transport into the β cell, glucose is metabolized to pyruvate via glycolysis and processed in the tricarboxylic acid (TCA) cycle in mitochondria. This increases the ATP/ADP ratio and causes the closure of ATP-dependent K^+^-channels in the plasma membrane. The resulting change in membrane potential modifies intracellular Ca^2+^, via voltage-gated Ca^2+^-channels, and triggers insulin exocytosis (Rorsman and Ashcroft, 2018). In addition to ATP, metabolic coupling factors, generated by metabolite cycles associated with the TCA cycle, amplify insulin secretion (Maechler et al., 2006).

Mitochondria in β cells are organized in a complex and dynamic network that continuously changes to adapt to the metabolic needs of the cell. This plasticity is due to a balance between fission and fusion of pre-existing mitochondria (Molina et al., 2009). In addition to these inter-mitochondrial changes, modification of the inner membrane structure and composition have also been observed in β cells in response to nutritional changes and oxidative stress and are supposed to control protons exchange and ATP production (Mannella, 2006).

Accumulating evidence indicates that mitochondrial dysfunction plays an important role in type 2 diabetes, a pathological condition that develops when β cells fail to release proper amounts of insulin in response to glucose, resulting in hyperglycemia and metabolic dysregulation (Fex et al., 2018). In type 2 diabetes, oxidative and glycolytic glucose metabolism is reduced, and mitochondria are characterized by high production of reactive oxygen species (ROS), which, under hyperglycemic conditions, are thought to exacerbate pathological pathways (Lowell and Shulman, 2005; Mulder and Ling, 2009; Haythorne et al., 2019).

These metabolic alterations in β cells could be related to altered mitochondrial dynamics, leading to impaired glucose-stimulated insulin secretion, as described by Rovira-Llopisa et al. (2017). They reported that type 2 diabetes inhibits mitochondrial oxygen consumption and alters mitochondrial fusion and fission processes.

Mitochondria are highly dynamic organelles that are able to move along microtubules and link to the actin network, which is involved in cellular responses to mechanical forces. Emerging evidence has demonstrated that the cytoskeleton and the extracellular mechanical factors are two important regulators of mitochondrial activity. Deformation of the cell during normal tissue function and the mechanical ECM stiffness influence mitochondrial morphology and ATP generation (Bartolák-Suki et al., 2017).

In keeping with these finding, our previous results regarding the outcome of mechanotransduction on human islets of Langerhans grown on cluster-assembled zirconia films (ns-ZrO_*x*_) prompted us to investigate the possible effect of nanostructures on mitochondria and their surroundings. However, islets represent an extremely complex model, they are composed of several cell populations that respond to different stimuli. Therefore, to gain insight into the molecular mechanism by which nanotopography may affect mitochondria morphology and function, we employed βTC3 cells, a mouse clonal β-cell line obtained by expression of the SV40 T antigen under control of the insulin promoter in transgenic mice (Efrat et al., 1988). The transformed cell line retains some characteristics of normal β cells, such as the ability to produce both proinsulin I and II and efficiently store them into secretory granules where the pro-hormone is processed to mature insulin.

Using the same proteomic approach described in Galli et al. (2018), i.e., shotgun proteomic for protein identification and label free for quantification, in this work, we focused our attention on the mitochondrial-enriched fraction of βTC3 cells grown on ns-ZrO_*x*_, in comparison to Glass and polycrystalline zirconia film with a very low roughness (flat Zirconia), and characterized the morphological and proteomic modifications induced by the nanostructure. Main results were also confirmed in the insulinoma INS1E β-cell line.

All together, the data suggest that βTC3 and INS1E cells sense substrate nanotopography and activate a mechanotransductive pathway involving modification of the mitochondrial activity and changes in the delicate balance between fusion and fission. These changes preserve β-cell homeostasis but yet at the same time allow a cellular response to the mechanical stimulus. Indeed, the nanostructure alters the inner mitochondrial membrane dynamics and the interplay with other organelles such as ER and lysosomes, thus resulting in a metabolic shift at the mitochondrial level.

## Materials and Methods

### Substrates Preparation

Nanostructured zirconia films with controlled and reproducible nanoscale morphology were produced by SCBD using a deposition apparatus equipped with a pulsed microplasma cluster source (PMCS), as described in detail in Piseri et al. (2001) and Schulte et al. (2016).

In the PMCS, an argon plasma jet ignited by a pulsed electric discharge ablates a zirconium rod. Zr atoms and ions sputtered from the target thermalize with the argon and traces of oxygen present in the condensation chamber and aggregate to form ZrO_*x*_ clusters. The mixture of clusters and inert gas then expands into a vacuum, through a nozzle, to form a seeded supersonic beam. The clusters carried by the seeded supersonic beam are collected on a substrate intersecting the beam trajectory (deposition rate of about 0.5–2.5 nm/min) and placed in a second vacuum chamber, thus forming a cluster-assembled film. Further oxidation of ZrO_*x*_ clusters takes place upon exposure to ambient atmosphere thus forming a ZrO_*x*_ film.

Four different batches of cluster-assembled ZrO_2_ films (called ns-ZrO_*x*_ hereafter) with roughness Rq of 10, 15, 20, and 25 nm were produced on round glass coverslips (Ø13 and Ø40 mm). As a reference we also produced flat ZrO_2_ films (Rq = 0.4 nm) by electron beam evaporation of a solid Zr target (flat-ZrO_2_). For the experiments, the samples with zirconia surfaces were sterilized with UV for 10 min directly before seeding the cells on them.

#### Cell Line and Culture Conditions

Mouse βTC3 (kindly provided by Prof. Douglas Hanahan, Department of Biochemistry and Biophysics, University of California, San Francisco, CA) and rat INS1E (kindly provided by Prof. Claes B. Wollheim, Department of Internal Medicine, University Medical Centre of Geneva, Geneva, Switzerland) cell lines were used. βTC3 cells were cultured in RPMI 1640 medium supplemented with 10% heat inactivated fetal bovine serum, 1% glutamine, and 1% penicillin-streptomycin (Di Cairano et al., 2011). INS1E cells were cultured in RPMI 1640 medium integrated with 10% heat inactivated fetal bovine serum, 1% glutamine, 1% penicillin-streptomycin, 1% HEPES-NaOH pH 7.4, 1% sodium pyruvate, and 50 μM β-mercaptoethanol. βTC3 and INS1E cells were seeded at density of 220 cells/mm^2^ onto glass coverslips, flat-ZrO_2_, and ns-ZrO_*x*_ substrates and were incubated in humidified atmosphere containing 5% of CO_2_ at 37°C. All the experiments reported in the work were performed in cells cultured for 3 days on different substrates.

#### Cell Viability

βTC3 cells were labelled with NucBlue^®^ Live and NucGreen^®^ Dead reagents (R37609, Invitrogen) following the manufacturer’s protocol, and cell viability was assessed by photographing random field at 40× magnification using the Axio Observer Z1 microscope (Zeiss). In order to quantify the percentage of dead cells over total cells, the number of NucBlue^®^ Live and NucGreen^®^ Dead positive cells was counted (AG and AM). Mean values and standard deviations were evaluated on the basis of three independent experiments.

#### Quantitative Immunofluorescence and TIRF Microscopy

βTC3 and INS1E cells were fixed and labeled with anti-vinculin antibody (1:75 – Sigma-Aldrich, V9131), TRITC-phalloidin (1:500 – Sigma Aldrich, P1951), and DAPI (1:10000 – Applichem, A1001). Random fields were imaged by epifluorescence (phalloidin and DAPI) and TIRF (vinculin) microscopy using a Carl Zeiss microscope, equipped with a 100 × 1.45 numerical aperture (NA) oil immersion objective and an Argon laser as the illumination source (Galli et al., 2018). Green fluorescence was excited using the 488-nm laser line and imaged with a band-pass filter (Zeiss) mounted on a Retiga SRV CCD camera. To quantify the dimension and shape of fluorescent objects, the Image-Pro Plus object analysis plug-in was used. The area (μm^2^) of vinculin-positive clusters and actin fibers, together with the nuclei aspect (major/minor axis), were measured in a software-assisted manner (Galli et al., 2018).

#### Intracellular ROS

Intracellular ROS were monitored using DCFDA (2’,7’-dichlorofuorescein diacetate – D6883, Sigma Aldrich), a membrane permeable probe fluorescent when tied to ROS. βTC3 cells were pre-loaded with 15 μM DCFDA in Krebs-Ringer Buffer (125 mM NaCl, 5 mM KCl, 1.2 mM MgSO_4_, 1.2 mM KH_2_PO_4_, 25 mM HEPES-NaOH pH 7.4 and 2 mM CaCl_2_) supplemented with 11 mM glucose at 37°C for 60 min. Reactive oxygen species production was quantified by fluorimetry with the microplate reader TECAN infinite^®^ F500 (485/528 nm Ex/Em). Mean values and standard deviations were evaluated on the basis of three independent experiments.

#### Mitochondrial Morphology

βTC3 and INS1E cells were labeled with 300 μM MitoSpy^TM^ Green FM (424805, Biolegend) in Krebs-Ringer Buffer supplemented with 11 mM glucose at 37°C for 30 min. Samples were positioned in an imaging chamber, and random fields were captured using the 488 nm filter of the Axio Observer Z1 microscope (Zeiss). To evaluate mitochondrial morphology, the following parameters were analyzed using the ImageJ particle analysis plug-in: area (μm^2^), aspect (major axis/minor axis), maximum feret (μm), and the number of mitochondria per cell. According to Stiles and Shirihai (2012), labeled structures with a diameter >17 μm were excluded from the analysis.

#### Mitochondrial Membrane Potential

Mitochondrial membrane potential was quantified by loading βTC3 cells with 100 nM MitoSpy^TM^ Orange CMTMRos (424803, Biolegend) in Krebs-Ringer Buffer supplemented with 11 mM glucose at 37°C for 30 min. Fluorescence intensity was detected with the microplate TECAN infinite^®^ F500 reader (551/576 nm Ex/Em). Experiments were performed in triplicate and data were expressed as fold increase over glass samples.

#### Evaluation of Contact Sites Between Mitochondria and Endoplasmic Reticulum

To mark the endoplasmic reticulum (ER), βTC3 and INS1E cells were transfected with an ER-GFP construct (GFP fused to the ER retention signal of calreticulin, a kind gift of Dr. Piccoli Giovanni, University of Trento, Italy) using Lipofectamine 3000 (L3000015, Invitrogen). Mitochondria were labeled with 100 nM MitoSpy^TM^ Orange CMTMRos (424803, Biolegend) by following the procedure described above. At 48 h after transfection cells were fixed using 4% PFA (paraformaldehyde) and random fields were captured using the 488 nm and the rhodamin filters of the Axio Observer Z1 microscope (Zeiss). To enhance the image quality prior to the colocalization analysis, the background was substracted and images were pre-processed using the “unsharp mask” filter. Images colocalization was evaluated by means of the ImageProPlus software.

#### Western Blotting

βTC3 and INS1E cells were collected and lysed in RIPA buffer (150 mM NaCl, 50 mM Tris HCl pH 7.6, 1 mM EDTA, 1% NP40, 0.5% Deoxicholate supplemented with aprotinin, PMSF, and Roche inhibitors) for 40 min at 4°C. Protein concentration was evaluated by Bradford assay (Sigma-Aldrich), and 15 μg of proteins were resolved by 10% SDS-PAGE and transferred onto nitrocellulose membranes (Millipore). Primary antibodies were applied for 2 h or overnight in blocking buffer (5% non-fat milk or 5% BSA in 150 mM NaCl, 20 mM Tris pH 7.4, and 0.1% Tween). The following primary antibodies were used: mouse anti-β actin 1:10000 (NB600501, Novus), rabbit anti-Mfn2 1:1000 (9482, Cell Signaling), rabbit anti-DRP1 1:1000 (8570, Cell Signaling), rabbit anti-OPA1 1:1000 (80471, Cell Signaling), rabbit anti-TOM20 1:1000 (42406, Cell Signaling), rabbit anti-P-eIF2α (Ser 51) 1:1000 (3597, Cell Signaling), rabbit anti-eIF2α 1:1000 (5324, Cell Signaling), and rabbit anti-PERK 1:1000 (3192, Cell Signaling). The secondary HRP-conjugated antibodies (Dako) were used at 1:5000 dilutions. For eIF2α, membranes were first stained with the antibody against the phosphorylated form of the protein, stripped and re-probed with the anti-protein antibody, as previously described (Galli et al., 2018). Proteins were detected by the ECL detection system (Cyanagen) using Odyssey Fc Image system and band density was quantified by Image Studio^TM^ Lite software (Li-Cor, Biosciences). Mean values and standard deviations were evaluated on the basis of three independent experiments performed in triplicate.

### Proteomic Analysis by Shotgun Mass Spectrometry and Label Free Quantification

#### Mitochondrial Enrichment

βTC3 cells were trypsinizated, lysed and the mitochondrial-enriched fraction was extracted with the Mitochondrial isolation kit (MITOISO2, Sigma–Aldrich) following the manufacturer’s protocol (Alberio et al., 2017).

#### Label-Free Shotgun Proteomics

After reduction and derivatisation, the proteins were digested with trypsin sequence grade (Roche) for 16 h at 37°C using a protein:trypsin ratio of 20:1 (Tedeschi et al., 2011; Maffioli et al., 2017).

LC-ESI-MS/MS analysis was performed on a Dionex UltiMate 3000 HPLC System with an EASY-Spray PepMap RSLC C18 column (150 mm, internal diameter of 75 μm) (Thermo Fisher Scientific). The gradients were 4% ACN in 0.1% formic acid for 3 min, 4–40% ACN in 0.1% formic acid for 90 min, 40–45% ACN in 0.1% formic acid for 10 min, 45–90% ACN in 0.1% formic for 16 min, and 90–94% for 14 min at a flow rate of 0.3 μl/min. The eluate was electrosprayed into an Orbitrap Fusion Tribrid (Thermo Fisher Scientific) through a nanoelectrospray ion source (Thermo Fisher Scientific). The Orbitrap Fusion Tribrid was operated in a positive data-dependent acquisition mode to automatically alternate between a full scan (300–1,500 m/z) in the Orbitrap (at resolution 120,000, AGC target 4,000,000) and subsequent HCD MS/MS in the Orbitrap of the 20 most intense peaks from the full scan (at resolution 15,000, normalized collision energy of 30%). The isolation window was 1.6 Da, unassigned charge state, rejected; charge state 1, rejected; charge states 2+, 3+, 4+, +5, +6, and +7, not rejected; and dynamic exclusion enabled, 30 s. Data acquisition was controlled by Xcalibur 4.1 and Tune 3.0 software (Thermo Fisher Scientific). The mass spectra were analyzed using MaxQuant software (version 1.6.0.1). The initial maximum allowed mass deviation was set to 6 ppm for monoisotopic precursor ions and 0.5 Da for MS/MS peaks. The enzyme specificity was set to trypsin, defined as C-terminal to arginine and lysine excluding proline, and a maximum of two missed cleavages were allowed. Carbamidomethyl cysteine was set as a fixed modification, N-terminal acetylation, methionine oxidation and asparagine/glutamine deamidation as variable modifications. The spectra were searched by the Andromeda search engine against the Homo Sapiens Uniprot sequence database (release June 3, 2019). The reversed sequences of the target database were used as a decoy database. Protein identification required at least one unique or razor peptide per protein group. The quantification in MaxQuant was performed using the built-in XIC-based label free quantification (LFQ) algorithm using fast LFQ (Vernocchi et al., 2014). The required false positive rate was set to 1% at the peptide and 1% at the protein level against a concatenated target decoy database, and the minimum required peptide length was set to seven amino acids. Statistical analyses were performed using the Perseus software (version 1.5.5.3)^1^. Only proteins present and quantified in at least two out of three repeats were considered as positively identified in a sample and used for statistical analyses.

##### Volcano plots

We performed the comparison between cells grown on nanostructured zirconia with the roughness Rq of 15 nm rms (ns-ZrO_*x*_) versus glass coverslips (Glass), cells grown on flat-ZrO_2_ versus ns-ZrO_*x*_ and Glass vs flat-ZrO_2_. In each comparison, proteins were considered differentially expressed if they were present only in one condition or showed significant *t*-test difference (*t*-test *p* ≤ 0.05). In the comparison Glass vs ns-ZrO_*x*_, 194 proteins are exclusively expressed or upregulated in ns-ZrO_*x*_, while 467 are exclusively expressed in Glass or downregulated in ns-ZrO_*x*_; in the comparison flat-ZnO_2_vs ns-ZrO_*x*_, 145 proteins are exclusively expressed or upregulated in ns-ZrO_*x*_, while 622 are exclusively expressed in flat-ZrO_2_ or downregulated in ns-ZrO_*x*_; and, in the comparison Glass vs flat-ZrO_2_, 439 proteins are exclusively expressed or upregulated in flat-ZrO_2_, while 231 are exclusively expressed in Glass or downregulated in flat-ZrO_2_.

ID lists were then searched by Mitominer, a mitochondrial localization database (Smith and Robinson, 2016) which integrates protein data from HomoloGene, Gene Ontology, KEGG, OMIM MS/MS, GFP (green fluorescent protein) localization data, and targeting sequence predictions, to obtain the entries annotated as mitochondrial in each data set. Only proteins with an Integrated Mitochondrial Protein Index (IMPI) ≥ 0.5 were considered mitochondrial molecules and were filtered out for further classification by bioinformatics software to cluster enriched annotation groups of GO Biological Processes (GOBP), Molecular Function (GOMF), Pathways and Networks by Panther software (Version 10.0; Toni et al., 2019), DAVID software (release 6.7; Toni et al., 2019), and STRING (Szklarczyk et al., 2019). Functional grouping was based on *p*-value ≤ 0.05, a DAVID enrichment score of three, and at least three counts.

The mass spectrometry proteomics data have been deposited to the ProteomeXchange Consortium via the PRIDE (Vizcaíno et al., 2016) partner repository with the dataset identifier PXD015739.

## Results and Discussion

### βTC3 Cells Sense Substrate Nanotopography and Activate a Mechanotransductive Pathway Involving Modification in Mitochondrial Activity

**FIGURE 1:**
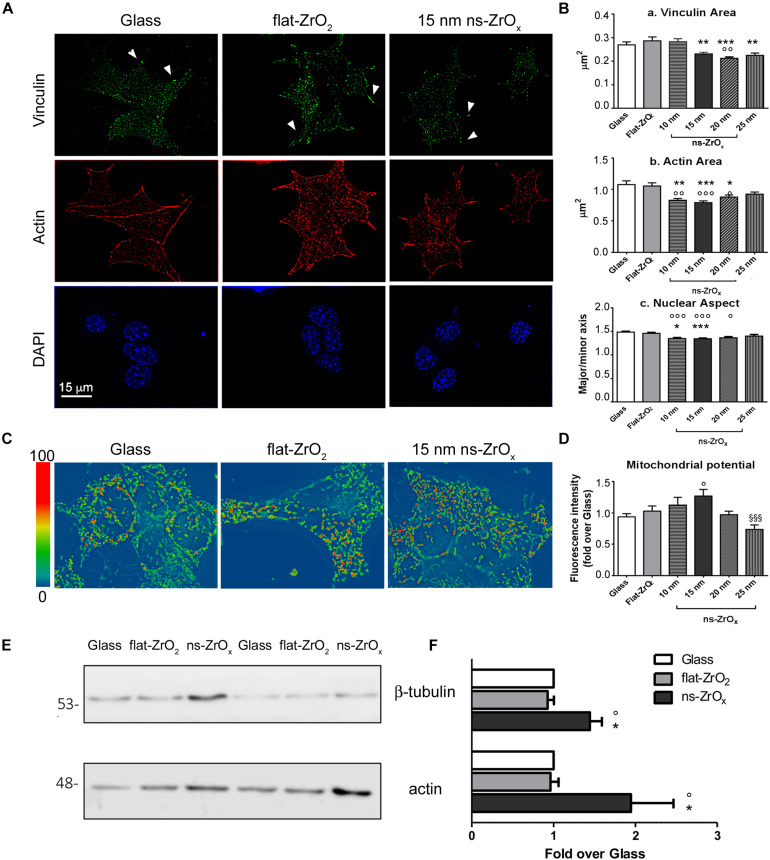
Nanostructured zirconia substrates promote the activation of a mechanotransduction pathway in βTC3 cells. **(A)** Cells grown on glass covers (Glass) as well as flat- (flat-ZrO_2_) or 15 nm nanostructured-zirconia (ns-ZrO_*x*_) substrates for 3 days were triple stained with anti-vinculin antibody (green), phalloidin (actin, red), and DAPI (blue). Representative epifluorescence (actin and DAPI) and TIRFM (vinculin) images are shown. Bar: 15 μm. Arrowheads indicate focal complexes. **(B)** Quantitative analyses of **(a)** vinculin-positive area, **(b)** cytoskeletal actin fibers area, and **(c)** nuclear architecture (major/minor axis) in cells grown on Glass, flat-ZrO_2_ and 10, 15, 20, 25 nm ns-ZrO_*x*_. Bars illustrate the average responses ± SE (*N* = 30–40 cells for each substrate, three independent experiments (*p < 0.05, **p < 0.01, ***p < 0.005, ns-ZrO_*x*_ vs flat-ZrO_2_; °*p* < 0.05, °°*p* < 0.01, °°°*p* < 0.005, ns-ZrO_*x*_ vs Glass). **(C)** Cells were loaded with MitoSpy^TM^ Orange CMTMRos and the mitochondrial membrane potential was measured by fluorimetry (551/576 nm Ex/Em). Representative images in pseudocolor are shown (blue low intensity, red high intensity). **(D)** Bars illustrate the average responses (fluorescence intensity) ± SE (*n* = 4 independent experiments in triplicate) (°*p* < 0.05 ns-ZrO_*x*_ vs Glass; ^§§§^ *p* < 0.005 25nm vs 15 nm ns-ZrO_*x*_). **(E)** Western-blotting analysis of β-tubulin and actin in βTC3 cells grown on the indicated substrates for three days (15 μg protein/sample). On the left, the protein molecular weight in kDa is reported. **(F)** The quantitative analysis shows the upregulation of cytoskeletal proteins expression in cells grown on 15 nm ns-ZrO_*x*_. Data (mean values ± S.D.; *n* = 5 independent experiments) are expressed as fold-change over Glass (*p < 0.05 ns-ZrO_*x*_ vs flat-ZrO_2_; °*p* < 0.05 ns-ZrO_*x*_ vs Glass).

We first verified whether βTC3 cells can sense changes in the ECM topography and activate a mechanotransduction pathway. Cells were seeded and cultured for 3 days on nanostructured (ns-ZrO_*x*_) substrates of different roughness (10, 15, 20, and 25 nm), and the organization of cell-substrate adhesions and actin cytoskeleton were analyzed by indirect immunofluorescence (Figure 1A). Flat zirconia (flat-ZrO_2_) substrates and glass coverslips were used as controls (Glass).

TIRF microscopy revealed the presence of vinculin-positive clusters distributed at the cell periphery in cells grown on glass covers and flat-ZrO_2_. On 10 and 15 nm ns-ZrO_*x*_ substrates, vinculin structures, probably nanoclusters or small focal contacts, were smaller in size and diffuse in cells (Figure 1Ba).

A similar trend was observed for actin filament structures (Figures 1A,Bb). Long actin fibers were mainly detected on glass covers and flat-ZrO_2_, but they seldom formed on 15 nm ns-ZrO_*x*_ where actin-labeled structures of reduced area were observed at the cell periphery and at cell–cell contact sites. Western blot analysis showed increased actin and tubulin expression in cells grown on nanostructured substrates compared to control covers, indicating a general reorganization of the cell cytoskeleton on ns-ZrO_*x*_ (Figures 1E,F).

It has been shown that mechanical forces, through reorganization of the actin cytoskeleton, also induced nuclear envelope modifications, chromatin architecture remodeling, and activation of a specific transcriptional program (Athirasala et al., 2017). In line with this possibility, quantitative analyses of DAPI staining revealed the presence of nuclear structures with increased roundness (major/minor axis) on 10 and 15 nm ns-ZrO_*x*_ compared to glass and flat-ZrO_2_ covers (Figure 1Bc).

In order to test whether the mechanotransduction pathway involves mitochondria, the organelle membrane potential was evaluated by MitoSpy^TM^ Orange, a dye whose concentration is related to the inner mitochondrial membrane potential (Figures 1C,D). Interestingly, cells grown on 15 nm ns-ZrO_*x*_ showed a statistically significant increase in membrane potential when compared to flat-ZrO_2_ and glass covers.

Taken together, these data confirm that βTC3 cells can sense the substrate nanotopography and activate a mechanotransductive pathway that involves not only cytoskeletal structures but also mitochondria. The most relevant effects were detected on 15 nm ns-ZrO_*x*_, therefore further analyses were performed on this substrate and results compared to those obtained with flat-ZrO_2_ and glass coverslips.

### Effect of Nanotopography on the Mitochondrial Proteome of βTC3 Cells

**FIGURE 2:**
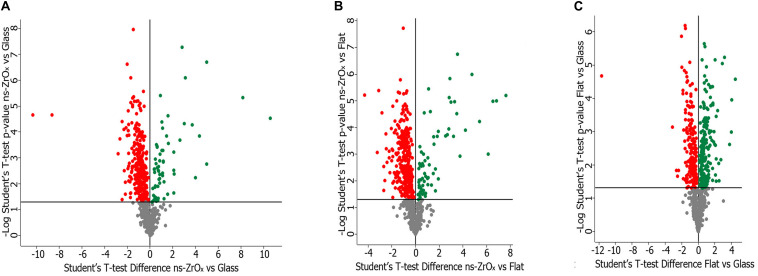
Volcano plot of the proteins differentially expressed in the comparison ns-ZrO_*x*_ vs Glass **(A)**, ns-ZrO_*x*_ vs flat-ZrO_2_ **(B)** and flat-ZrO_2_ vs Glass **(C)**. Proteins were considered differentially expressed if they were present only in one condition or showed significant *t*-test difference (*t*-test *p* ≤ 0.05). The proteins up- or downregulated are indicated in green and red, respectively.

To understand at molecular level the impact of the nanostructure on mitochondria and on their interplay with other organelles, such as lysosomes and the ER, the samples were analyzed by a label-free shotgun proteomic approach upon sub-fractionation to enrich in the mitochondrial components. Specific analyses were carried out by comparing (1) cells grown on glass coverslips (Glass) versus cells grown on ns-ZrO_*x*_, (2) cells grown on flat-ZrO_2_ versus cells grown on ns-ZrO_*x*_, and (3) cells grown on Glass vs cells grown on flat-ZrO_2_. Figure 2 shows the corresponding Volcano plots reporting the proteins differentially expressed. Almost 28% of all the proteins identified in each comparison are classified as mitochondria according to Mitominer. Non-mitochondrial proteins were also identified, mainly localized in compartments tightly associated to mitochondria (such as the ER, Golgi apparatus, and vacuole) in line with what reported in the literature using this enrichment method (Alberio et al., 2017).

The lists of mitochondrial proteins exclusively expressed in each growth condition or differently expressed (common protein whose expression is statistically significant different according to the *t*-test; *p*-value ≤ 0.05) in the three comparisons are shown in the Supplementary Material (Supplementary Tables S1–S6). These data sets were further analyzed in terms of GO classification and pathways.

#### The Nanostructure Modulates Proteins Involved in Mitochondrial Dynamic and Morphology

**FIGURE 3:**
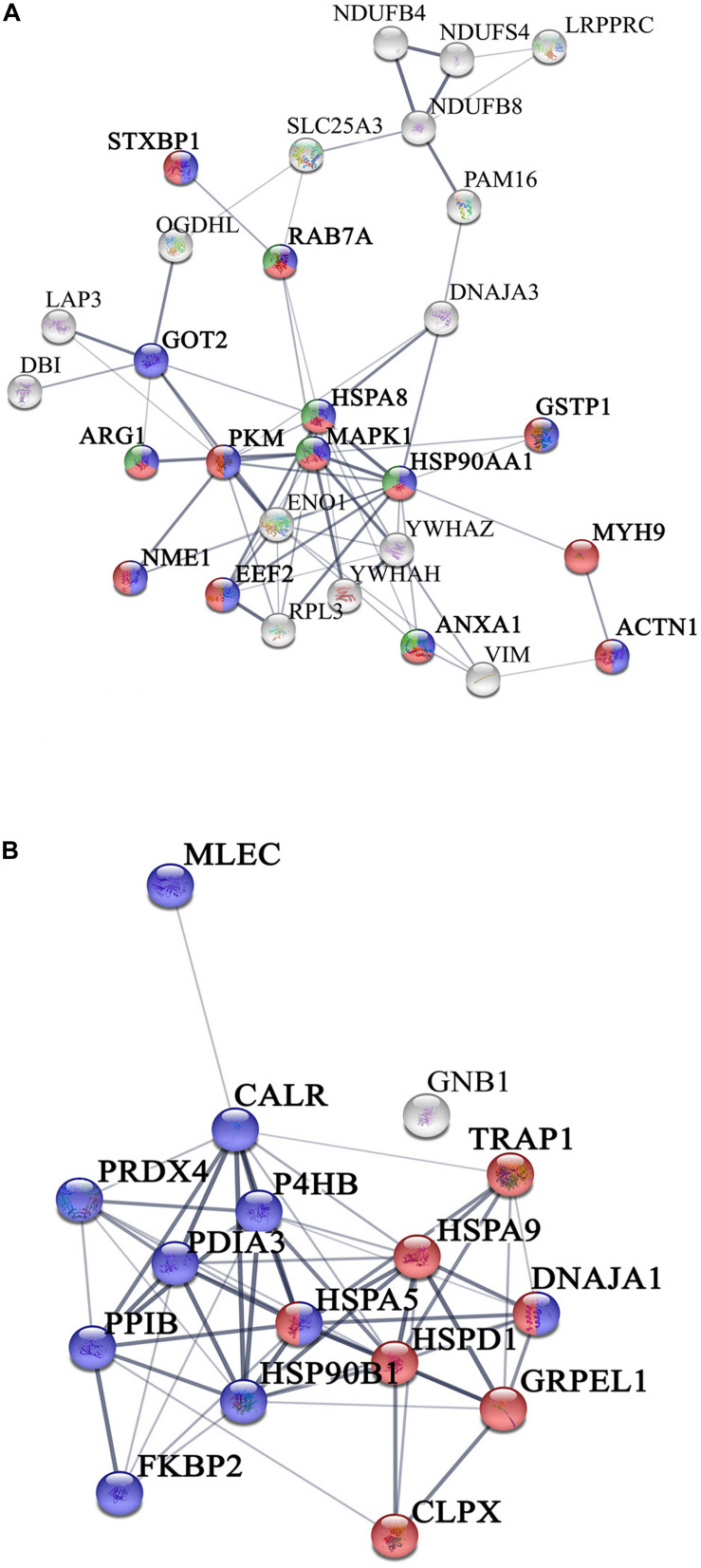
Network interactions of the proteins differentially expressed in the comparison Glass vs ns-ZrO_*x*_. **(A)** Proteins up regulated or only expressed in ns-ZrO_*x*_ and **(B)** proteins downregulated in ns-ZrO_*x*_ or exclusively expressed in Glass. Data sets were analyzed by String considering active interactions: text mining, experiments, and databases; edge thickness indicates “confidence.” The proteins are indicated by the official gene symbol. Nodes are colored to highlight the more significant enrichment classification. **(A)** Proteins upregulated or only expressed in ns-ZrO_*x*_ involved in vesicle mediated transport (red), secretion (blue), and lysosome transport (green). **(B)** Network of proteins down regulated on ns-ZrO_*x*_ or only expressed on Glass involved in protein localization, folding, and processing in mitochondria (red) and ER (blue).

We have previously demonstrated that, human islets of Langerhans grown on ns-ZrO_*x*_, in comparison with islets grown on gelatin, face a complex reorganization guided by mechanotransduction (Galli et al., 2018). In the present work we addressed the possible effect of the nanostructure on βTC3 cells focusing mainly on the same comparison, ns-ZrO_*x*_ vs Glass, but analyzing the mitochondrial enriched proteome (Figure 3).

The bioinformatic analysis strongly suggests that the nanostructure has a profound impact on the mitochondrial inner membrane lowering the expression of many proteins involved in cristae formation (Supplementary Tables S2, S7) (ATP5A1, ATP5B, ATP5C1, ATP5D, ATP5H, CHCHD3, IMMT, LETM1, and SAMM50) and cristae shaping (MICOS 19, 60, 10), these modifications are probably linked to the decrease in the expression of the ROS modulator 1 (ROMO1) and the optic atrophy 1 (OPA1; Supplementary Table S2, S7).

ROMO1 is a redox-regulated protein required for mitochondrial fusion and normal cristae morphology (Cogliati et al., 2016), while OPA1 promotes the inner membrane fusion and governs the delicate balance between fusion and fission in the dynamic mitochondrial network. A disturbance of this balance, often observed under stress and pathologic conditions, causes mitochondrial fragmentation and can ultimately result in cell death (Bartolák-Suki et al., 2017). Our data suggest that, to escape this event, cells responding to the nanostructure perturbation decrease also the expression of dynamin-1 like protein (DNM1L), involved in apoptosis and necrosis, and ganglioside-induced differentiation-associated protein 1 (GDAP1), which regulates the mitochondrial network by promoting mitochondrial fission (Supplementary Table S2; Bartolák-Suki et al., 2017; Tilokani et al., 2018).

**FIGURE 4:**
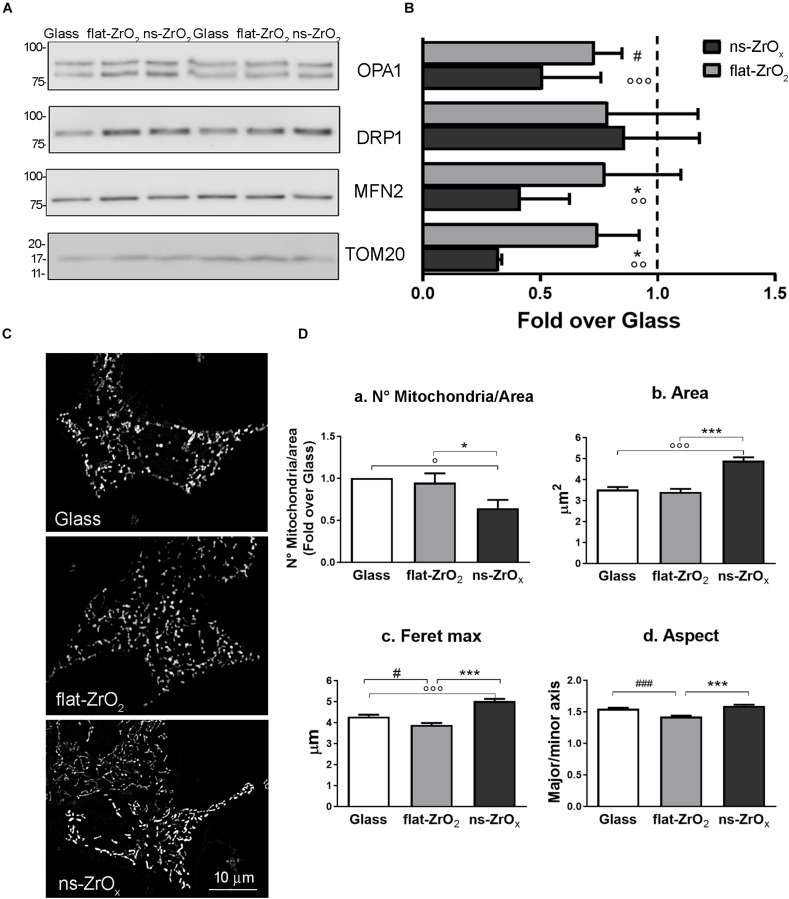
ns-ZrO_*x*_ substrates modify mitochondrial morphology. **(A)** Western-blotting analysis of mitochondrial pro-fission (DRP1), pro-fusion (MFN2 and OPA1) and import (TOM20) proteins in βTC3 cells grown on the indicated substrates for 3 days (15 μg protein/sample). On the left, the protein molecular weight in kDa is reported. **(B)** The quantitative analysis shows the downregulation OPA1, MFN2 and TOM20 expressions in cells grown on 15 nm ns-ZrO_*x*_. Data (mean values ± SD; *n* = 5 independent experiments) are expressed as fold-change over Glass (*p < 0.05 ns-ZrO_*x*_ vs flat-ZrO_2_; °°*p* < 0.01, °°°*p* < 0.005 ns-ZrO_*x*_ vs Glass; #*p* < 0.05 flat-ZrO_2_ vs Glass). **(C)** Cells grown on the indicated substrates for 3 days were loaded with MitoSpy^TM^ Green FM and imaged by epifluorescence microscopy. Representative images are shown. Bar: 10 μm. **(D)** The quantitative analyses of mitochondrial morphology are shown: **(a)** mitochondria number per area, **(b)** single mitochondria area (μm^2^), **(c)** feret maximum (μm), and **(d)** mitochondria aspect (major/minor axis) in cells grown on different substrates. Bars illustrate the average responses ± SE (*N* = 15-20 cells for each substrate in three independent experiments). (*p < 0.05, ***p < 0.005 ns-ZrO_*x*_ vs flat-ZrO_2_; °*p* < 0.05, °°°*p* < 0.005 ns-ZrO_*x*_ vs Glass; ^#^*p* < 0.05, ^###^*p* < 0.005 flat-ZrO_2_ vs Glass).

Data were confirmed by immunoblotting analysis of proteins involved in mitochondrial fusion and fission namely OPA1, mitofusin 2 (MNF2) and dynamin-related protein 1 (DRP1) (Figures 4A,B). In agreement with proteomic data, we found a significant decrease in the expression of both OPA1 and MFN2 proteins in cells grown on ns-ZrO_*x*_ compared to flat-ZrO_2_ and glass cover, no significant change in DRP1 was detected. Fusion and fission events therefore seem to be counterbalanced in our system ensuring cell homeostasis.

Recent finding report that mitochondrial cristae shape is strictly related to oxidative phosphorylation and that membrane morphology modulates this event, with a direct impact on cellular metabolism (Cogliati et al., 2016). A fused mitochondrial network allows matrix component distribution and stimulation of aerobic respiratory activity, while mitochondrial fragmentation predominates during elevated stress levels and cell death (Tilokani et al., 2018).

In βTC3 cells the mitochondrial network revealed by MitoSpy^TM^ Green FM, an organic dye which selectively binds mitochondrial proteins, was different among cells grown on different substrates and elongated mitochondria were often observed in cells grown on ns-ZrO_*x*_ (Figure 4C). The quantitative analyses confirmed the observations: cells grown on the nanostructure showed an increased average mitochondrial area (Figure 4Db), maximum diameter (Feret max) (Figure 4Dc) and aspect (major/minor axis) (Figure 4Dc) compared to those observed on both glass and flat-ZrO_2_ covers. In addition, the number of mitochondria per area (Figure 4Da) was significantly lower in cells grown on the nanostructured substrates when compared to control substrates, probably because they tend to organize in networks.

We confirmed main findings on nanostructure-induced modifications of the mitochondrial proteome and morphology in INS1E, a different cell line commonly used to model β-cell physiopathology (Wollheim, 2000). Supplementary Figure S1 showed that also INS1E cells sensed the substrate nanotopography and accordingly modified the actin cytoskeleton organization and the nuclear shape (Supplementary Figures S1A,B). INS1E mitochondria are generally more elongated and elaborated than those observed in the mouse model, but, exactly as reported for βTC3 cells, their average area (Supplementary Figure S2Db) and maximum diameter (Feret max) (Supplementary Figure S2Dc) increased when cells were grown on the nanostructure compared to glass and flat-ZrO_2_ coverslips. Furthermore, western blotting experiments confirmed also in the INS1E line the downregulation of MFN2 and TOM20 in cells grown on ns-ZrO_*x*_ compared to control flat substrates (Supplementary Figures S2A,B).

Taken together, the cellular and proteomic data suggest that the nanostructure modulates the delicate balance between fusion and fission, preserving β-cell homeostasis but concomitantly allowing a cellular response to the mechanical stimulus. The mitochondria remodeling triggered by the nanostructure is similar in the βTC3 and INS1E cell lines, suggesting that it can be a specific response of β-cells to the nanotopography.

#### The Nanostructure Leads to a Metabolic Shift at Mitochondrial Level

**FIGURE 5:**
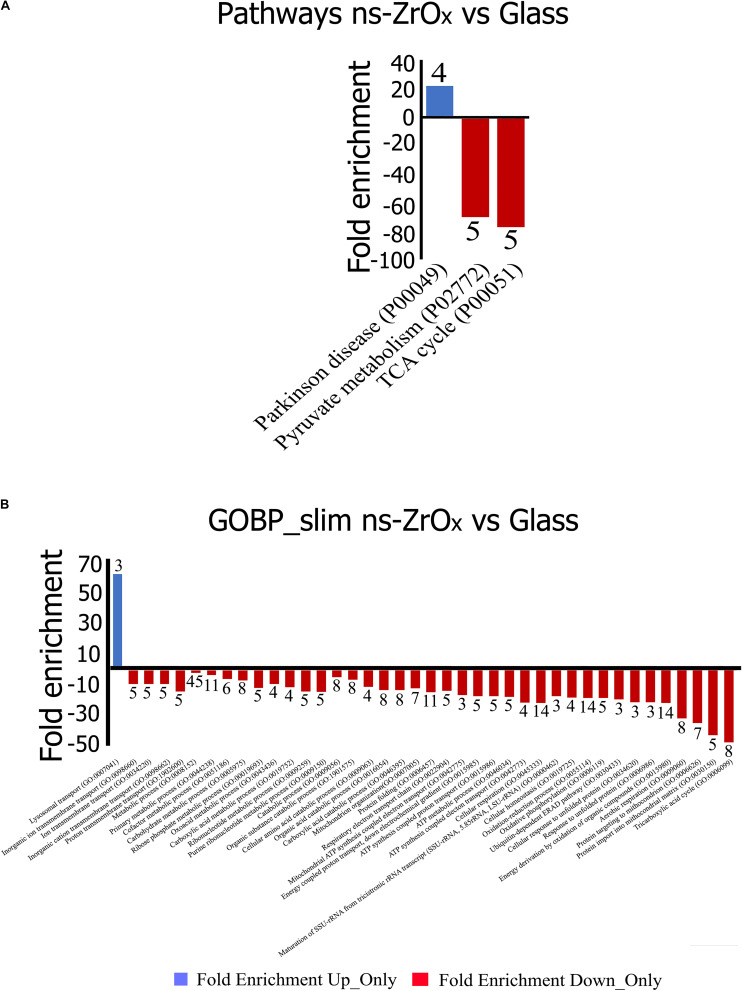
Gene Ontology classification of proteins differentially expressed in the comparison Glass vs ns-ZrO_*x*_. The differentially expressed proteins were classified into different Pathways **(A)** and Biological Processes (GOBP-slim) **(B)** using the Panther software. Functional grouping was based on *p*-value ≤ 0.05 and minimum three counts. Negative values (Red) refer to fold enrichment of proteins less expressed at ns-ZrO_*x*_ or only expressed in Glass whereas positive value (Blue) refer to proteins more or only expressed on ns-ZrO_*x*_ in the comparison Glass vs ns-ZrO_*x*_. The numbers on the bars represent the counts.

From the data available in the literature, cristae emerge as structures that mediate the localization of oxidative phosphorylation complexes, creating a specialized and highly dynamic compartment that undergoes massive remodeling, with a direct impact on the energetic state of the cell (Cogliati et al., 2016). Moreover, mitochondrial-shaping proteins, such as OPA1, have emerged as potential modulators of mitochondrial bioenergetics. In accordance with the effect on mitochondrial dynamic and morphology described above, the proteomic data highlight that the nanostructure hampers the oxidative metabolism and the expression of proteins involved in TCA cycle, pyruvate metabolism, aerobic respiratory and ATP synthesis coupled to proton transport (Figure 5, Table 2, and Supplementary Table S7) probably through the regulation of actin cytoskeleton (ACTN1, MAPK1, and MYH9) (Figures 1D,E, 5, Table 2 and Supplementary Table S7). Table 3 reports the list of all the proteins down regulated in cells grown on ns-ZrO_*x*_ or exclusively expressed in Glass that are involved in the TCA cycle and are complex of the aerobic respiratory chain.

**TABLE 1:** DAVID functional grouping in terms of KEGG Pathways of the mitochondrial proteins differentially expressed in cells grown in ns-ZrO_*x*_ in comparison with cells grown on Glass.

**Increased or only expressed in ns-ZrO_*x*_**				
**DAVID KEGG Pathway**				
**Term**	**Count**	***p*-Value**	**Genes**	**Fold Enrich.**

Biosynthesis of antibiotics	7	1.7E-04	GOT2, PKM, ARG1, NME1, OGDHL, ALDH9A1, ENO1	7.8
Arginine and proline metabolism	4	1.0E-03	GOT2, LAP3, ARG1, ALDH9A1	19.0
Viral carcinogenesis	6	1.3E-03	PKM, MAPK1, YWHAZ, YWHAH, ACTN1, DNAJA3	6.9
Biosynthesis of amino acids	4	3.0E-03	GOT2, PKM, ARG1, ENO1	13.2
Alzheimer’s disease	5	4.4E-03	MAPK1, NDUFB4, NDUFS4, NDUFB8, IDE	7.1
Carbon metabolism	4	1.0E-02	GOT2, PKM, OGDHL, ENO1	8.4
Metabolic pathways	11	1.8E-02	GOT2, PKM, LAP3, ARG1, NDUFB4, NDUFS4, NDUFB8, NME1, OGDHL, ALDH9A1, ENO1	2.1
Glycolysis/Gluconeogenesis	3	3.0E-02	PKM, ALDH9A1, ENO1	10.6

**Decreased in ns-ZrO_*x*_ or only expressed in Glass**				
**DAVID KEGG Pathway**				

**Term**	**Count**	***p*-value**	**Genes**	

Citrate cycle (TCA cycle)	16	8.7E-22	SUCLG2, SUCLG1, CS, IDH3B, ACLY, OGDH, PCK2, PDHB, IDH3A, SDHA, DLD, IDH2, PDHA1, MDH2, PC, FH	42.7
Parkinson’s disease	18	8.3E-13	NDUFA4, ATP5D, SLC25A5, SLC25A6, ATP5B, NDUFA13, UQCRQ, VDAC3, NDUFA12, VDAC1, SDHA, NDUFV1, NDUFV2, ATP5C1, COX6B1, ATP5A1, ATP5H, NDUFS2	10.1
Metabolic pathways	41	3.7E-10	ATP5D, ALDH18A1, GANAB, GLUD1, ATP5B, NFS1, OGDH, UQCRQ, PDHB, IVD, IDH2, COX6B1, PDHA1, RPN2, HADH, NDUFS2, ATP5H, FH, NDUFA4, SUCLG2, SUCLG1, CS, MAOB, NDUFA13, IDH3B, ACLY, PCK2, NDUFA12, IDH3A, SDHA, DHRS4, NNT, NDUFV1, NDUFV2, DLD, ATP5C1, ATP5A1, PCCB, OAT, MDH2, PC	2.7
Ribosome	15	8.2E-10	RPL13, RPLP2, RPS15A, RPS8, RPS18, MRPL12, RPS16, RPL23, RPL6, RPL34, RPS14, RPL8, RPL5, RPL4, RPL10A	8.8
Oxidative phosphorylation	14	6.6E-09	NDUFA4, SDHA, ATP5D, NDUFV1, ATP5B, NDUFV2, COX6B1, ATP5C1, NDUFA13, ATP5A1, UQCRQ, NDUFS2, ATP5H, NDUFA12	8.4
Alzheimer’s disease	14	1.1E-07	NDUFA4, SDHA, ATP5D, NDUFV1, ATP5B, NDUFV2, COX6B1, ATP5C1, NDUFA13, ATP5A1, UQCRQ, NDUFS2, ATP5H, NDUFA12	6.7
Pyruvate metabolism	7	8.2E-06	DLD, PDHA1, PCK2, MDH2, PDHB, PC, FH	14.0
Protein processing in ER	10	2.2E-04	HYOU1, P4HB, HSP90B1, GANAB, PDIA3, RRBP1, DNAJA1, HSPA5, RPN2, CALR	4.7
Oxocarboxylic acid metabolism	4	1.1E-03	CS, IDH2, IDH3B, IDH3A	18.8
Biosynthesis of amino acids	6	1.9E-03	ALDH18A1, CS, IDH2, IDH3B, IDH3A, PC	6.7
Glyoxylate and dicarboxylate metabolism	4	4.3E-03	DLD, CS, PCCB, MDH2	11.9
Valine, leucine and isoleucine degradation	4	2.0E-02	IVD, DLD, HADH, PCCB	6.8
Propanoate metabolism	3	4.6E-02	SUCLG2, SUCLG1, PCCB	8.6
Glycolysis/Gluconeogenesis	4	5.0E-02	DLD, PDHA1, PCK2, PDHB	4.8

*The Table reports the proteins statistically differentially expressed among the two different conditions (*t*-test *p* ≤ 0.05). The column “Counts” indicate the number of genes present in each category. Functional grouping was based on *p* ≤ 0.05 and at least three counts.*

**TABLE 2:** DAVID functional grouping of the mitochondrial proteins down regulated in ns-ZrO_*x*_ or exclusively expressed on Glass in the comparison ns-ZrO_*x*_ vs Glass.

**DAVID Clustering**				
**Term**	**Count**	***p*-Value**	**Genes**	**Fold Enrich.**

**Annotation Cluster 1 Enrichment Score: 17.5**				
Citrate cycle (TCA cycle)	16	8.7E-22	SUCLG2, SUCLG1, CS, IDH3B, ACLY, OGDH, PCK2, PDHB, IDH3A, SDHA, DLD, IDH2, PDHA1, MDH2, PC, FH	42.7
tricarboxylic acid cycle	14	6.2E-21	SUCLG2, SUCLG1, CS, IDH3B, OGDH, PDHB, IDH3A, SDHA, NNT, DLD, IDH2, PDHA1, MDH2, FH	64.9
**Annotation Cluster 2 Enrichment Score: 10.9**				
SRP-dependent cotranslational protein targeting to membrane	14	1.9E-13	RPL13, RPLP2, RPS15A, RPS8, RPS18, RPL23, RPS16, RPL6, RPL34, RPS14, RPL8, RPL5, RPL4, RPL10A	20.0
structural constituent of ribosome	18	6.5E-13	RPL13, SLC25A5, SLC25A6, RPS15A, RPLP2, RPS8, RPS18, MRPL12, SLC25A25, RPS16, RPL23, RPL6, RPL34, RPS14, RPL8, RPL5, RPL4, RPL10A	10.9
Ribosomal protein	16	7.8E-13	RPL13, RPLP2, RPS15A, RPS8, RPS18, MRPL12, RPS16, RPL23, RPL6, RPL34, RPS14, RPL8, RPL5, MRPL48, RPL4, RPL10A	13.6
translation	18	5.1E-12	RPL13, RRBP1, SLC25A5, SLC25A6, RPS15A, RPLP2, RPS8, RPS18, SLC25A25, RPS16, RPL23, RPL6, RPL34, RPS14, RPL8, RPL5, RPL4, RPL10A	9.6
**Annotation Cluster 3 Enrichment Score: 9.3**				
Parkinson’s disease, Huntington’s disease	18	8.3E-13	NDUFA4, ATP5D, SLC25A5, SLC25A6, ATP5B, NDUFA13, UQCRQ, VDAC3, NDUFA12, VDAC1, SDHA, NDUFV1, NDUFV2, ATP5C1, COX6B1, ATP5A1, ATP5H, NDUFS2	10.1
Oxidative phosphorylation	14	6.6E-09	NDUFA4, SDHA, ATP5D, NDUFV1, ATP5B, NDUFV2, COX6B1, ATP5C1, NDUFA13, ATP5A1, UQCRQ, NDUFS2, ATP5H, NDUFA12	8.4
**Annotation Cluster 4 Enrichment Score: 5.7**				
nucleotide phosphate-binding region: FAD	7	2.9E-06	SDHA, GPD2, IVD, AIFM1, DLD, ETFDH, ETFA	17.4
**Annotation Cluster 5 Enrichment Score: 5.1**				
mitochondrial electron transport, NADH to ubiquinone	7	1.5E-06	NDUFA4, NDUFV1, NDUFV2, DLD, NDUFA13, NDUFS2, NDUFA12	19.2
**Annotation Cluster 6 Enrichment Score: 4.6**				
mitochondrial proton-transporting ATP synthase	6	2.8E-07	ATP5D, USMG5, ATP5B, ATP5C1, ATP5A1, ATP5H	40.7
**Annotation Cluster 7 Enrichment Score: 3.8**				
Pyruvate	5	3.4E-06	DLD, PDHA1, PCK2, PDHB, PC	46.2
Glycolysis/Gluconeogenesis	4	5.0E-02	DLD, PDHA1, PCK2, PDHB	4.8
**Annotation Cluster 8 Enrichment Score: 3.0**				
protein import into mitochondrial matrix	4	2.5E-04	GRPEL1, TIMM17A, TOMM20, TIMM23B	31.6
∼P-P-bond-hydrolysis-driven protein transmembrane transporter activity	3	1.5E-03	TIMM17A, TOMM20, TIMM23B	50.2

*The Table reports the proteins down regulated in ns-ZrO_*x*_ or exclusively expressed on Glass (*t*-test *p* ≤ 0.05). The column “Counts” indicates the number of genes present in each category. Functional grouping was based on *p* ≤ 0.05 and at least three counts.*

**TABLE 3:** List of all the mitochondrial proteins differentially regulated in the comparison ns-ZrO_*x*_ vs Glass that are involved in TCA cycle and the complex of the aerobic respiratory chain.

**Classification**		**Gene ID**	**Protein name**	
TCA Cycle		PDHB	Pyruvate dehydrogenase E1 component subunit beta, mitoch.	↓
		SUCLG1	Succinate-CoA ligase [ADP/GDP-forming] sub.alpha mitoch	↓
		OGDH	2-oxoglutarate dehydrogenase, mitochondrial	↓
		IDH3A	Isocitrate dehydrogenase [NAD] subunit alpha, mitochondrial	↓
		CS	Citrate synthase	↓
		IDH3B	Isocitrate dehydrogenase [NAD] subunit beta, mitochondrial	↓
		PDHA1	Pyruvate dehydr. E1 comp. sub, alpha, somatic form, mitoch.	↓
		MDH2	Malate dehydrogenase, mitochondrial	↓
		SDHA	Succinate dehydr. [ubiqu,] flavoprot. subunit, mitochondrial	↓
		SUCLG2	Succinate-CoA ligase [GDP-forming] subunit beta, mitochon.	↓
		FH	Fumarate hydratase, mitochondrial	↓
		MDH1	Malate dehydrogenase, cytoplasmic	↓
		ACLY	ATP citrate lyase	↓
		DLD	dihydrolipoamide dehydrogenase	↓
		IDH2	isocitrate dehydrogenase (NADP(+)) 2	↓
		IDH3G	isocitrate dehydrogenase (NAD(+)) 3 non-catalytic sub. gamma	↓
		OGDHL	oxoglutarate dehydrogenase like	↑
		PC	pyruvate carboxylase	↓
		PCK2	phosphoenolpyruvate carboxykinase 2, mitochondrial	↓
		SUCLA2	succinate-CoA ligase ADP-forming beta subunit	
Mitochondrial respiratory chain	complex I	NDUFB8	NADH dehydr.[ubiq.] 1 beta subcomplex subunit 8, mitoch.	↑
	complex I	SLC25A3	Phosphate carrier protein, mitochondrial	↑
	complex I	NDUFA12	NADH dehydr. [ubiquinone] 1 alpha subcomplex subunit 12	↓
	complex I	NDUFV2	NADH dehydr. [ubiquinone] flavoprotein 2, mitochondrial	↓
	complex I	NDUFA13	NADH dehydr. [ubiquinone] 1 alpha subcomplex subunit 13	↓
	complex I	NDUFS2	NADH dehydr. [ubiquinone] iron-sulfur protein 2, mitoch.	↓
	NADH:ubiq core subunit	NDUFS8	NADH:ubiquinone oxidoreductase core subunit S8	↑
		NDUFV1	NADH:ubiquinone oxidoreductase core subunit V1	↓
	NADH:ubiq. supernumerary subunits	NDUFB4	NADH:ubiquinone oxidoreductase subunit B4	↑
		NDUFS4	NADH:ubiquinone oxidoreductase subunit S4	↑
	complex II	SDHA	Succinate dehydr. [ubiquinone] flavoprotein subunit, mitoch	↓
	complex III	UQCRQ	Cytochrome b-c1 complex subunit 8	↓
	complex IV	COA3	Cytochrome c oxidase assembly factor 3 homolog, mitocho.	↓
	complex IV	COX6B1	Cytochrome c oxidase subunit 6B1	↓
	complex IV	NFUFA4	Cytochrome c oxidase subunit NDUFA4	↑
	complex IV	NDUFA4	Cytochrome c oxidase subunit NDUFA4	↓
	complex IV	MT-CO2	mitochondrially encoded cytochrome c oxidase II	↓
	complex V	ATP5B	ATP synthase subunit beta, mitochondrial	↓
	complex V	ATP5C1	ATP synthase subunit gamma, mitochondrial	↓
	complex V	ATP5A1	ATP synthase subunit alpha, mitochondrial	↓
	complex V	ATP5D	ATP synthase subunit delta, mitochondrial	↓
	Transporter IN	SLC25A5	ADP/ATP translocase 2	↓

*All the proteins down regulated in cells grown on ns-ZrO_*x*_ or exclusively expressed in Glass are listed in Supplementary Table S2. Table 3 reports only the proteins of Supplementary Table S2 that are described as involved in TCA cycle and the complex of the aerobic respiratory.*

Concomitantly, the nanostructure triggers the higher expression of the glycolytic enzyme enolase (ENO1) as well as arginase1 (ARG1; Table 1 and Supplementary Table S1). The latter is involved in the homeostasis of L-arginine in competition with nitric oxide synthase (NOS) that utilizes the intracellular substrate arginine for NO synthesis, a possible source of ROS production. In accordance, in the comparison Glass vs ns-ZrO_*x*_ we observed the decrease of oxidative stress response proteins (TXN2, PRDX3, PARP1, AIFM1, ETFDH, NDUFA12, NDUFS2, PARP1, PRDX3, PRDX5, ROMO1, and TXN2) (Table 2 and Supplementary Tables S2, S7) associated to an increase in Glutathione S-transferase P (GSTP1) (Supplementary Table S1) that may protect cells from oxidative stress.

The general picture, however, does not suggest a hypoxic condition. Although ENO1, which is regulated by the hypoxia-inducible factor 1 alpha protein (HIF1α), is increased in cells grown on ns-ZrO_*x*_, our previous results demonstrated that the nanostructure triggers the expression of hypoxia-inducible factor prolyl hydroxylase 2 (PHD2) considered to be the main HIF1α levels regulator. Indeed, under normoxia, PHD2 hydroxylates HIF1α proline residues, marking it for subsequent ubiquitination and proteasomal degradation (Meneses and Wielockx, 2016). In accordance, the mitochondrial fraction of cells grown on ns-ZrO_*x*_ shows a lower expression of hypoxia upregulated protein 1 (HYOU1) as well as a decrease in the apoptosis-inducing factor 1 (AIFM1) and the mitochondrial outer-membrane voltage-dependent anion channel 1 (VDAC1), which plays a central role in regulating metabolism and apoptosis (Supplementary Table S2), suggesting that the metabolic effect observed is not linked to hypoxic conditions and/or apoptotic events.

**FIGURE 6:**
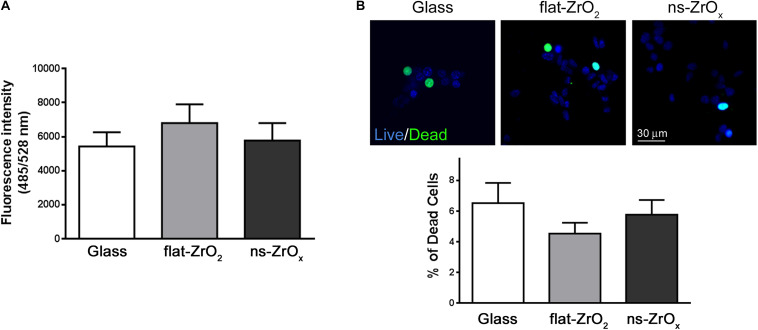
Nanostructure impact on ROS content and βTC3 cell viability. **(A)** ROS content. Intracellular ROS were monitored by DCFDA and ROS production was quantified by fluorimetry (485/528 nm Ex/Em). Data are expressed as mean ± SD of three independent experiments. **(B)** Cell viability. Representative images of cells stained with NucBlue^®^ (Live cells) and NucGreen^®^ (Dead cells). Bar, 30 μm. Bars represent the percentage of dead cells over the total cells. Data are expressed as mean ± SD of three independent experiments (a minimum of 100 cells for experiment was counted).

In line with proteomic results, the ROS content is similar in cells grown on different substrates (Figure 6A) and cell viability is preserved in cells maintained on ns-ZrOx substrates (Figure 6B).

#### Nanostructure Alters the Inner Mitochondrial Membrane Dynamics and the Interplay With Organelles

The expression of the most abundant import machineries of proteins into the mitochondrial inner membrane is altered by the nanostructure that decreases the mitochondrial import receptor subunit TOM20 homolog (TOMM20) and the mitochondrial import inner membrane translocases subunit (TIMM17A, TIMM23B) as well as the sorting and assembly machinery component 50 homolog (SAMM50), thus hampering the inner mitochondrial membrane organization (Tables 1, 2, and Supplementary S7 and Figure 3B). Immunoblotting analysis confirmed the downregulation of TOM20 in both βTC3 and INS1E lines (Figures 4A,B and Supplementary Figures S2A,B).

Among the proteins upregulated by the substrate roughness there are proteins involved in vesicle-mediated transport (HSP90AA1, HSPA8, MYH9, RAB7A, YWHAH, and YWHAZ) and lyososomal transport (XSPA8, STXBP1, and RAB7A) (Figures 3A, 5 and Supplementary Table S7).

Particularly interesting is Ras-related protein Rab-7a (RAB7A), a protein known to regulate the lysosomal dynamic, the traffic between late endosomes and lysosomes, and the formation of mitochondria-lysosome membrane contact sites (Hutagalung and Novick, 2011). These contacts mark sites of mitochondrial fission, allowing regulation of mitochondrial networks by lysosomes, whereas conversely, mitochondrial contacts regulate lysosomal RAB7 hydrolysis and dynamics via TBC1 domain family member 15 (TBC1D15; Wong et al., 2018), which, however, is not increased or altered in our data sets. Mitochondria and lysosome have been shown to actively interact upon cellular stress and play a role in mitochondrial degradation via mitophagy and in other degradative events, but these organelles can also directly interact via non-degradative processes through the dynamic formation of inter-organelle membrane contact sites in healthy cells (Wong et al., 2019). The contact sites have been found to be important for mediating multiple cellular functions, including the regulation of mitochondrial division and the transfer of lipids, calcium, and iron (Hutagalung and Novick, 2011).

Concomitantly, the expression of proteins involved in protein localization, folding and processing in ER is decreased in ns-ZrO_*x*_ (CALR, CLPX, DNAJA1, FKBP2, GNB1, GRPEL1, HSP90B1, HSPA5, HSPA9, HSPD1, MLEC, P4HB, PDIA3, PPIB, PRDX4, and TRAP1) (Table 1, 2 and Supplementary Table S7). All are membrane proteins playing a pivotal role in protein folding and, most of them, are involved in response to stress and apoptotic processes (CALR, PDIA3. P4HB, GNB1, HSPA5, HSP90B1, HSPD1, and TRAP1).

As shown in Figure 3B, some of these proteins are also described in the mitochondria (CRPX, GRPEL1, HSPD1, DNAJA1, HSPA5, HSPA9, and TRAP1) suggesting that the nanostructure alters a protein network involved in the complex interplay between ER and mitochondria. This is not surprising since almost 20% of the mitochondrial surface is in close apposition with the ER (Naon and Scorrano, 2014).

**FIGURE 7:**
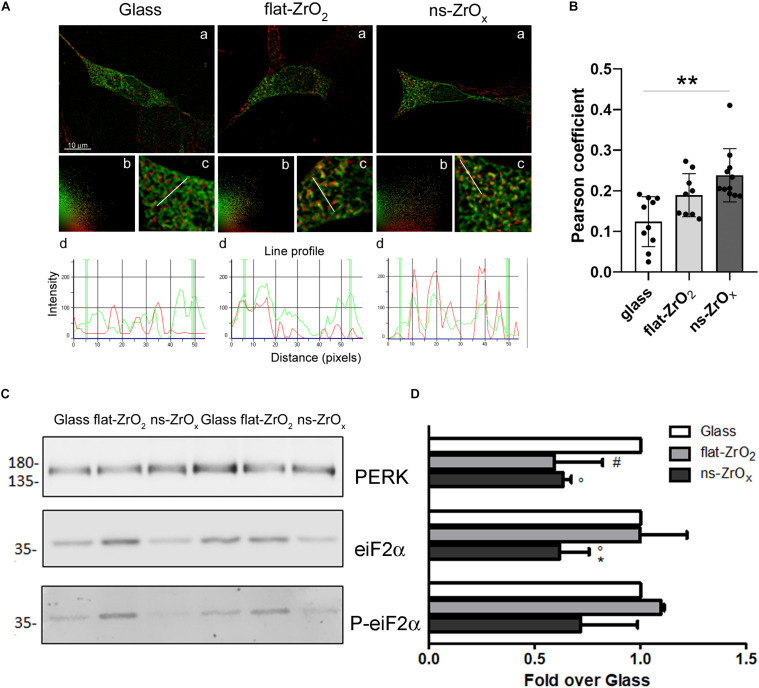
Nanostructure impact on ER-mitochondrial network organization and UPR response. **(A)** βTC3 cells, plated on the different substrates, were transfected with ER-GFP (green) and, after 48 h, the mitochondria were labeled with MitoSpy^TM^ Orange (red). **(a)** Representative images of ER-mitochondrial networks in cells grown on the indicated substrates. The yellow/orange staining highlights ER-mitochondria juxtaposition. Bar, 10 μm. **(b)** Relative scatter plot analysis of red and green stainings; **(c)** particular of panel *a* at higher magnification (3X); and **(d)** plot of red and green fluorescence intensities along the line profile reported in *c*. **(B)** Pearson’s colocalization coefficient. Data are expressed as mean ± SE. Single values are reported. **p < 0.01 vs Glass. **(C)** Western-blotting analysis of eIF2α, P-eIF2α and PERK in cells grown on the indicated substrates for 3 days (15 μg protein/sample). **(D)** The quantitative analysis of protein expression shows downregulation of PERK and eIF2α in cells grown on 15 nm ns-ZrO_*x*_. Data (mean values ± SD; *n* = 3 independent experiments) are expressed as fold-change over Glass (*p < 0.05 ns-ZrO_*x*_ vs flat-ZrO_2_; °*p* < 0.05 ns-ZrO_*x*_ vs Glass; ^#^*p* < 0.05 flat-ZrO_2_ vs Glass).

In order to confirm modifications of the ER-mitochondria network, βTC3 and INS1E cells grown on the three different substrates were transfected with an ER-GFP construct selectively targeted to the ER compartment and mitochondria were labeled with the MitoSpy^TM^ Orange. Intricated ER cisternae networks spanning the whole cellular volume were clearly detectable in high magnification images (Figure 7Ac), and no apparent differences in their organization were observed in cells grown on different substrates. Mitochondria of similar size were visible in all samples, but only in cells grown on the nanostructure they were clearly tethered to the ER. Observations are corroborated by the Pearsons’ colocalization coefficient analysis which reveals increased ER–mitochondria juxtaposition in cells grown on the nanostructure compared to glass covers in βTC3 cells; similar results were observed in INS1E cells (Figures 7A,B and Supplementary Figures S3A,B).

Mitochondria and ER are two metabolic organelles physically and functionally interconnected, sharing some important cellular functions and playing a key role in the control of cellular homeostasis (Madreiter-Sokolowski et al., 2019). It is well known that mitochondrial dysfunction and ER stress are involved in the development of type 2 diabetes and perturbation of their crosstalk could also participate to the development of this disease (Rieusset, 2011). Although proteomic results showed a modified ER-mitochondria crosstalk on the nanostructure, we did not detect sign of ER stress. On the contrary, the expression of Eukaryotic Initiation Factor 2 alpha (eIF2α), its phosphorylated form (P-eIF2α), and protein kinase RNA-like ER kinase (PERK) was significantly decreased in cells grown on ns-ZrO_*x*_ compared to flat Zirconia and Glass cover, indicating that the Unfolded Protein Response was not activated on the nanostructure (Figures 7C,D), again supporting an healthy state of the cell.

Interestingly the measured lengths of tethers between the mitochondrial external membrane and smooth ER are 9–16 nm, and those between mitochondria-lysosome contact sites have an average distance of ∼10 nm in the range of the 15 nm roughness of the Zirconia substrate utilized in our experiments. We suggest that the substrates roughness may create a pattern of peaks and valley that can be sensed by cells and may act as constrains, forcing cytoplasmic components and organelles to get closer and to interact with each other’s. We also speculate that modulation of the ER-organelles interactions induced by the nanostructure can be involved in the effect observed on βTC3 cells.

## Conclusion

In conclusion, proteomic and functional data strongly suggest that mitochondria morphology and proteome is directly regulated by the nanotopography. The major differences were observed in the mitochondrial inner membrane domain; indeed, we found a decreased expression of many proteins involved in cristae formation and shaping. In turn, this modification probably drags the decrease in TCA cycle and aerobic respiration observed in cells grown on nanosubstrates. Immunoblotting and morphological studies revealed same modifications in two distinct beta cell lines (βTC3 and INS1E), thus suggesting that it may be a general mechanism of beta cell adaptation to changes in substrate topography (Galli et al., 2020).

A similar modification of mitochondrial activity has been recently observed in a mouse model of eulipidaemic diabetes (βV59M diabetic mice). Transcriptomics and proteomics data obtained in islet of these mice show up regulation of proteins involved in glycolysis/gluconeogenesis and down regulation of those involved in oxidative phosphorylation. Glucose-induced increases in NADH and ATP are impaired, and both oxidative and glycolytic glucose metabolism are reduced, indicating that hyperglycemia markedly reduces mitochondrial metabolism and ATP synthesis; this consequently modifies β-cells metabolism (Haythorne et al., 2019).

However, there are also important differences. Indeed, while we observed decreased expression of proteins involved in the TCA cycle, the mitochondrial membrane potential is higher in cells grown on the nanostructure, and no signs of apoptosis or ER stress are evident on this substrate. Therefore, we favor the idea that the morphological change induced in mitochondria by the nanostructure can prime mitochondria for a metabolic switch necessary to match the new needs of the cell. The proteomic and functional modification reported in this work probably represents only the initial stage of this adaptation since experiments were performed in 3-day-old cells. Further work aimed at monitoring during the time the adaptation of β-cell metabolism to the nanostructure will be necessary to understand the complex interplay between cells and the ECM.

To the best of our knowledge, we demonstrated for the first time that not only nutritional and oxidative stress but also mechanical forces can shape mitochondria structure and function, thus driving a metabolic switch in these cells.

## Data Availability Statement

The datasets generated for this study can be found in the ProteomeXchange Consortium with the dataset identifier PXD015739.

## Author Contributions

CPe, CL, and GT conceived the project. EM, AG, CPe, and GT wrote the principal part of the manuscript and realized the figures. EM, SN, AN, and GT contributed to the proteomic approach and related data analyses. AG and AM contributed to the cell culture, biochemical, and fluorescence experiments. CL, CPi, and PM executed the fabrication of the nanostructured surfaces by SCBD and flat-ZrO_2_ by electron beam evaporation. CL, CPe, and GT participated in the project conception/creation and the realization of the manuscript, also contributing reagents, materials, and analysis tools. All authors contributed to the article and approved the submitted version.

## Conflict of Interest

The authors declare that the research was conducted in the absence of any commercial or financial relationships that could be construed as a potential conflict of interest. The handling editor declared a past co-authorship with one of the authors GT.

## Footnotes

1 https://maxquant.net/perseus/

## Supplementary Material

FIGURE S1 **(A)** Representative epifluorescence images of INS1E cells grown on glass covers (Glass), flat- (flat-ZrO_2_) or 15 nm nanostructured-zirconia (ns-ZrO_*x*_) substrates for 3 days, doubled stained with phalloidin (actin, red) and DAPI (blue). Bar: 15 μm. **(B)** Quantitative analyses of cytoskeletal actin fibers (a) area and (b) length and nuclear (c) area and (d) architecture (major/minor axis) in cells grown on Glass, flat-ZrO_2_ and ns-ZrO_*x*_ are shown. Bars illustrate the average responses ± SE (^∗^*p* < 0.05 ns-ZrO_*x*_ vs flat-ZrO_2_; *p* < 0.05, *p* < 0.005 ns-ZrO_*x*_ vs Glass; #*p* < 0.05 flat-ZrO_2_ vs Glass).

FIGURE S2 Mitochondria proteome and morphology in INS1E cells. **(A)** Western-blotting analysis of mitochondrial pro-fission (DRP1), pro-fusion (MFN2 and OPA1) and import (TOM20) proteins in INS1E cells grown on the indicated substrates for 3 days (15 μg protein/sample). On the left, the proteins molecular weight in kDa is reported. **(B)** The quantitative analysis shows the downregulation MFN2 and TOM20 expressions in cells grown on 15 nm ns-ZrO_*x*_. Data (mean values ± SD; *n* = 3 independent experiments) are expressed as fold-change over Glass (*p* < 0.01 ns-ZrO_*x*_ vs Glass; ^##^*p* < 0.01 flat-ZrO_2_ vs Glass). **(C)** Cells grown on the indicated substrates for 3 days, were loaded with MitoSpy^TM^ Green FM and imaged by epifluorescence microscopy. Representative images are shown. Bar: 10 μm. **(D)** The quantitative analyses of mitochondrial morphology in cells grown on different substrates is shown: (a) mitochondria number per area, (b) single mitochondria area (μm^2^), (c) Feret maximum (μm), and (d) mitochondria aspect (major/minor axis). Bars illustrate the average responses ± SE (°°p < 0.01 ns-ZrO_*x*_ vs Glass; ^#^*p* < 0.05 flat-ZrO_2_ vs Glass).

FIGURE S3 ER-Mitochondrial networks. INS1E plated on glass, flat-ZrO_2_ or ns-ZrO_*x*_ substrates were transfected with ER-GFP (green) and after 48 h, the mitochondria were labeled with MitoSpy^TM^ Orange (red). **(A)** (a) Representative images of ER-mitochondrial networks in cells grown on the indicated substrates. The yellow/orange staining highlights ER-mitochondria juxtaposition. Bar 10 μm. (b) Scatter plot analysis of image a. (c) Particular of panel a at higher magnification (3X). (d) plot of red and green fluorescence intensities along the line profile reported in c. **(B)** Bars report Pthe average Pearson’s colocalization coefficient value ± SE. Single values are reported. ^∗^*p* < 0.05 vs Glass.

TABLE S1 Proteins up regulated or exclusively expressed in mitochondria of cells grown on ns-ZrO_*x*_ in the comparison ns-ZrO_*x*_ vs Glass. Proteins were considered differentially expressed if they were present only in one condition or showed a statistically significant difference (*t*-test *p* ≤ 0.05).

TABLE S2 Proteins down regulated in mitochondria of cells grown on ns-ZrO_*x*_ or exclusively expressed on Glass in the comparison ns-ZrO_*x*_ vs Glass. Proteins were considered differentially expressed if they were present only in one condition or showed a statistically significant difference (*t*-test *p* ≤ 0.05).

TABLE S3 Proteins up regulated or exclusively expressed in mitochondria of cells grown on ns-ZrO_*x*_ in the comparison ns-ZrO_*x*_ vs flat-ZrO_2_. Proteins were considered differentially expressed if they were present only in one condition or showed a statistically significant difference (*t*-test *p* ≤ 0.05).

TABLE S4 Proteins down regulated in mitochondria of cells grown on ns-ZrO_*x*_ or exclusively expressed on flat-ZrO_2_ in the comparison ns-ZrO_*x*_ vs flat-ZrO_2_. Proteins were considered differentially expressed if they were present only in one condition or showed a statistically significant difference (*t*-test *p* ≤ 0.05).

TABLE S5 Proteins up regulated or exclusively expressed in mitochondria of cells grown on flat-ZrO_2_ in the comparison flat-ZrO_2_ vs Glass. Proteins were considered differentially expressed if they were present only in one condition or showed a statistically significant difference (*t*-test *p* ≤ 0.05).

TABLE S6 Proteins down regulated in mitochondria of cells grown on flat-ZrO_2_ or exclusively expressed on Glass in the comparison flat-ZrO_2_ vs Glass. Proteins were considered differentially expressed if they were present only in one condition or showed a statistically significant difference (*t*-test *p* ≤ 0.05).

TABLE S7 STRING functional grouping of the proteins differentially expressed in mitochondria of cells grown on ns-ZrO_*x*_ in comparison with mitochondria of cells grown on Glass. Grouping is based on proteins that are statistically and differentially expressed among the two different conditions (*t*-test *p* ≤ 0.05) or present only in one condition. The column “Counts” indicates the number of genes present in each category. Functional grouping was based on FDR ≤ 0.05 and at least three counts.

BLOT S1 βTC3 cells grown on the indicated substrates were lysed and 15 μg of proteins were resolved by 10% SDS-PAGE and transferred onto nitrocellulose membrane. **(A)** Representative image of ponceau staining; the molecular weight in kDa is reported on the right. **(B)** The membrane was divided into 3 parts: the upper part (>58 kDa) upper part was used to evaluate the expression of OPA1, MFN2, and DRP1—; the middle (30–58 kDa) and the lower (<30 kDa) parts to quantify actin and TOM20 expressions, respectively.

BLOT S2 βTC3 cells grown on the indicated substrates were lysed and 15 μg of proteins were resolved by 10% SDS-PAGE and transferred onto nitrocellulose membrane. **(A)** Representative image of ponceau staining; the molecular weight in kDa is reported on the right. **(B)** The membrane was divided into 3 parts the lower part (< 48 kDa) was used to evaluate the expression of eIF2α and P-eIF2α, and the middle (48–100 kDa) and the upper (> 100 kDa) parts to quantify β-tubulin and PERK expressions, respectively.

BLOT S3 INS1E cells grown on the indicated substrates were lysed and 15 μg of proteins were resolved by 10% SDS-PAGE and transferred onto nitrocellulose membrane. **(A,C)** Representative images of ponceau staining. **(B,D)** The membranes were divided into 3 parts: the upper parts (> 58 kDa) were used to evaluate the expression of OPA1, MFN2, and DRP1, the middle (30–58 kDa) and the lower (< 30 kDa) parts to quantify actin and TOM20 expressions, respectively.
