# Supplementary figures and images for: Proteomic Analysis Reveals a Mitochondrial Remodeling of βTC3 Cells in Response to Nanotopography
Source: Front Cell Dev Biol. 2020 Jul 29;8:508. doi: 10.3389/fcell.2020.00508 (PMC7405422; doi:10.3389/fcell.2020.00508)

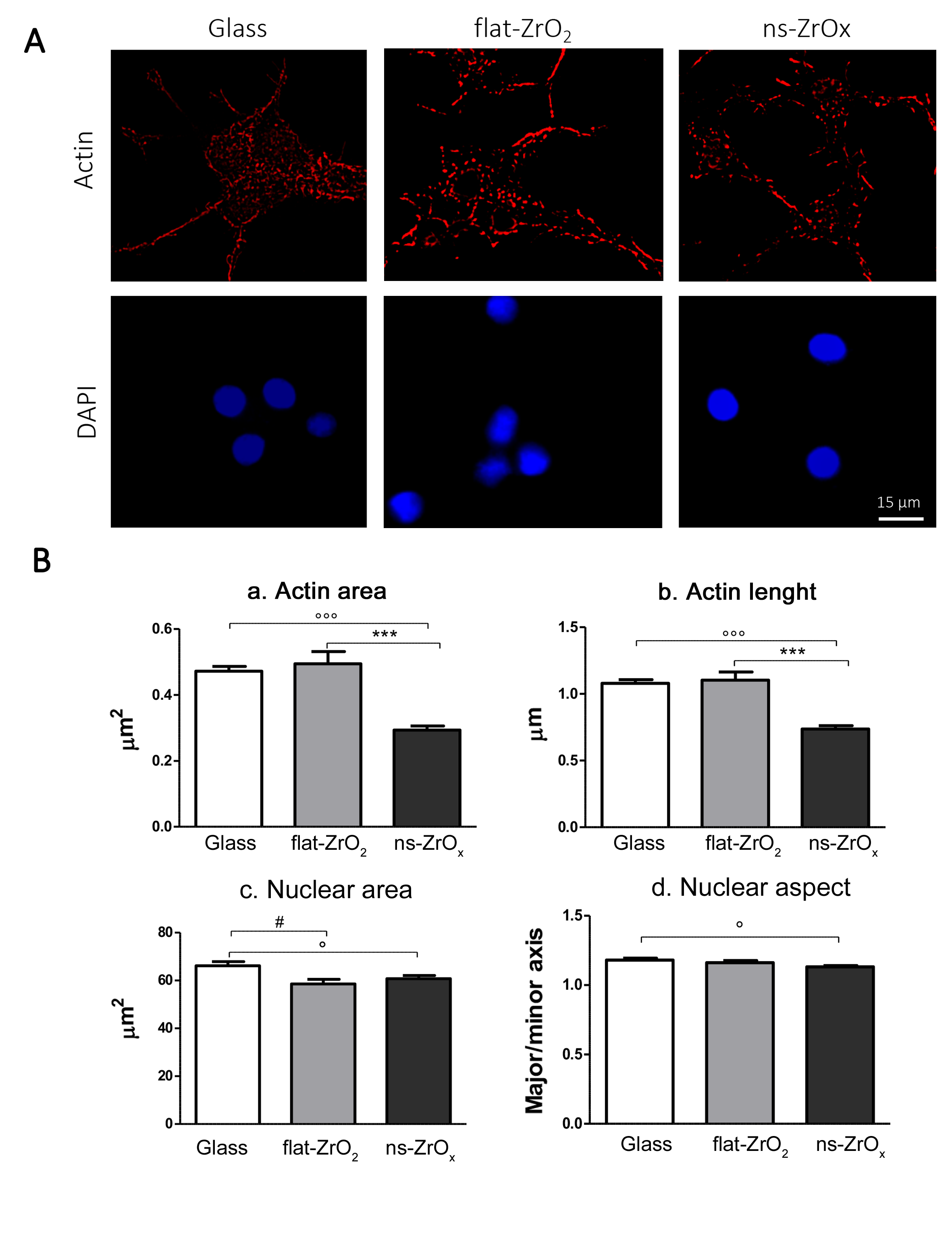

Supplement: FIGURE S1 — (A) Representative epifluorescence images of INS1E cells grown on glass covers (Glass), flat- (flat-ZrO2) or 15 nm nanostructured-zirconia (ns-ZrOx) substrates for 3 days, doubled stained with phalloidin (actin, red) and DAPI (blue). Bar: 15 μm. (B) Quantitative analyses of cytoskeletal actin fibers (a) area and (b) length and nuclear (c) area and (d) architecture (major/minor axis) in cells grown on Glass, flat-ZrO2 and ns-ZrOx are shown. Bars illustrate the average responses ± SE (∗p < 0.05 ns-ZrOx vs flat-ZrO2; p < 0.05, p < 0.005 ns-ZrOx vs Glass; #p < 0.05 flat-ZrO2 vs Glass). [file Image_1.tif]

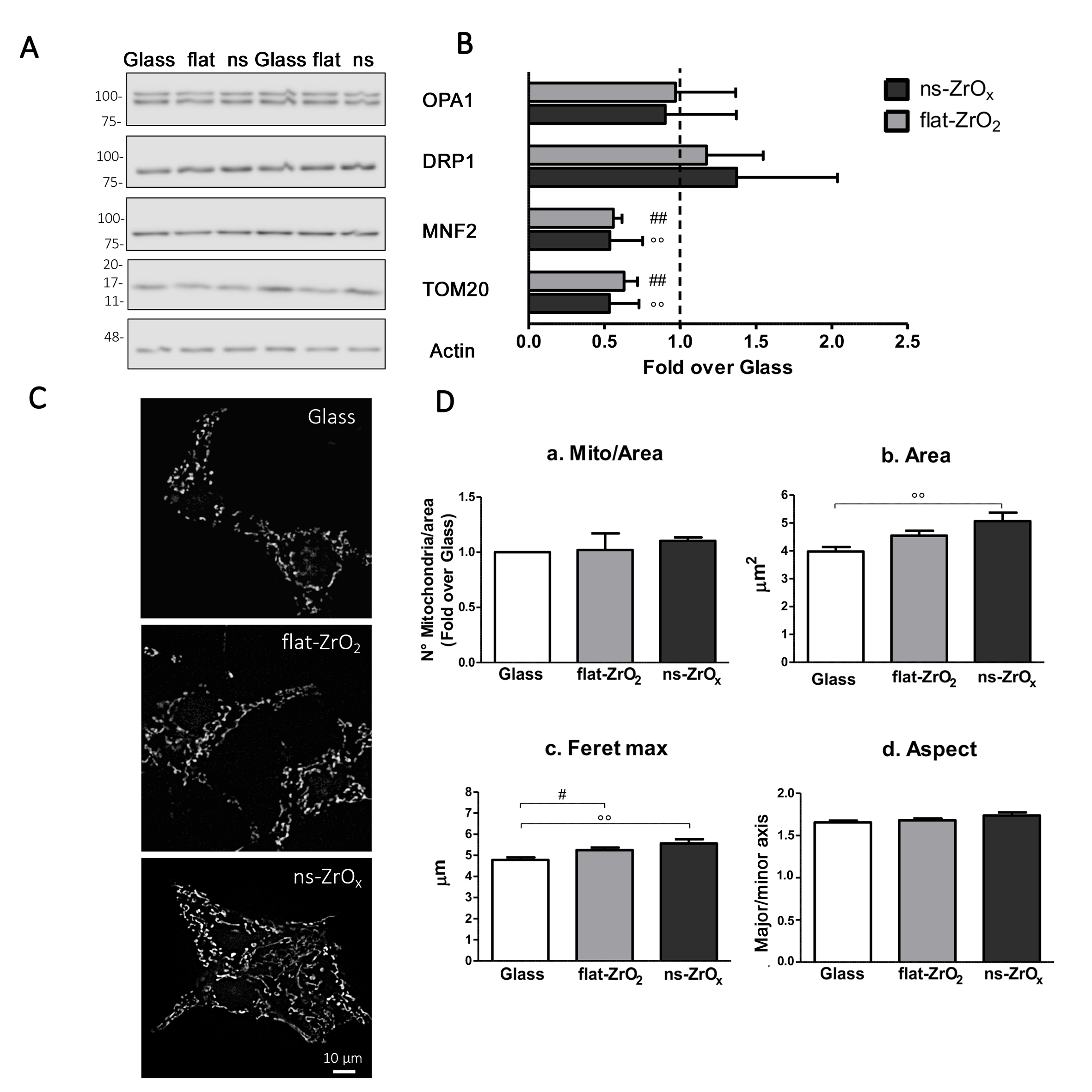

Supplement: FIGURE S2 — Mitochondria proteome and morphology in INS1E cells. (A) Western-blotting analysis of mitochondrial pro-fission (DRP1), pro-fusion (MFN2 and OPA1) and import (TOM20) proteins in INS1E cells grown on the indicated substrates for 3 days (15 μg protein/sample). On the left, the proteins molecular weight in kDa is reported. (B) The quantitative analysis shows the downregulation MFN2 and TOM20 expressions in cells grown on 15 nm ns-ZrOx. Data (mean values ± SD; n = 3 independent experiments) are expressed as fold-change over Glass (p < 0.01 ns-ZrOx vs Glass; ##p < 0.01 flat-ZrO2 vs Glass). (C) Cells grown on the indicated substrates for 3 days, were loaded with MitoSpyTM Green FM and imaged by epifluorescence microscopy. Representative images are shown. Bar: 10 μm. (D) The quantitative analyses of mitochondrial morphology in cells grown on different substrates is shown: (a) mitochondria number per area, (b) single mitochondria area (μm2), (c) Feret maximum (μm), and (d) mitochondria aspect (major/minor axis). Bars illustrate the average responses ± SE (°°p < 0.01 ns-ZrOx vs Glass; #p < 0.05 flat-ZrO2 vs Glass). [file Image_2.tif]

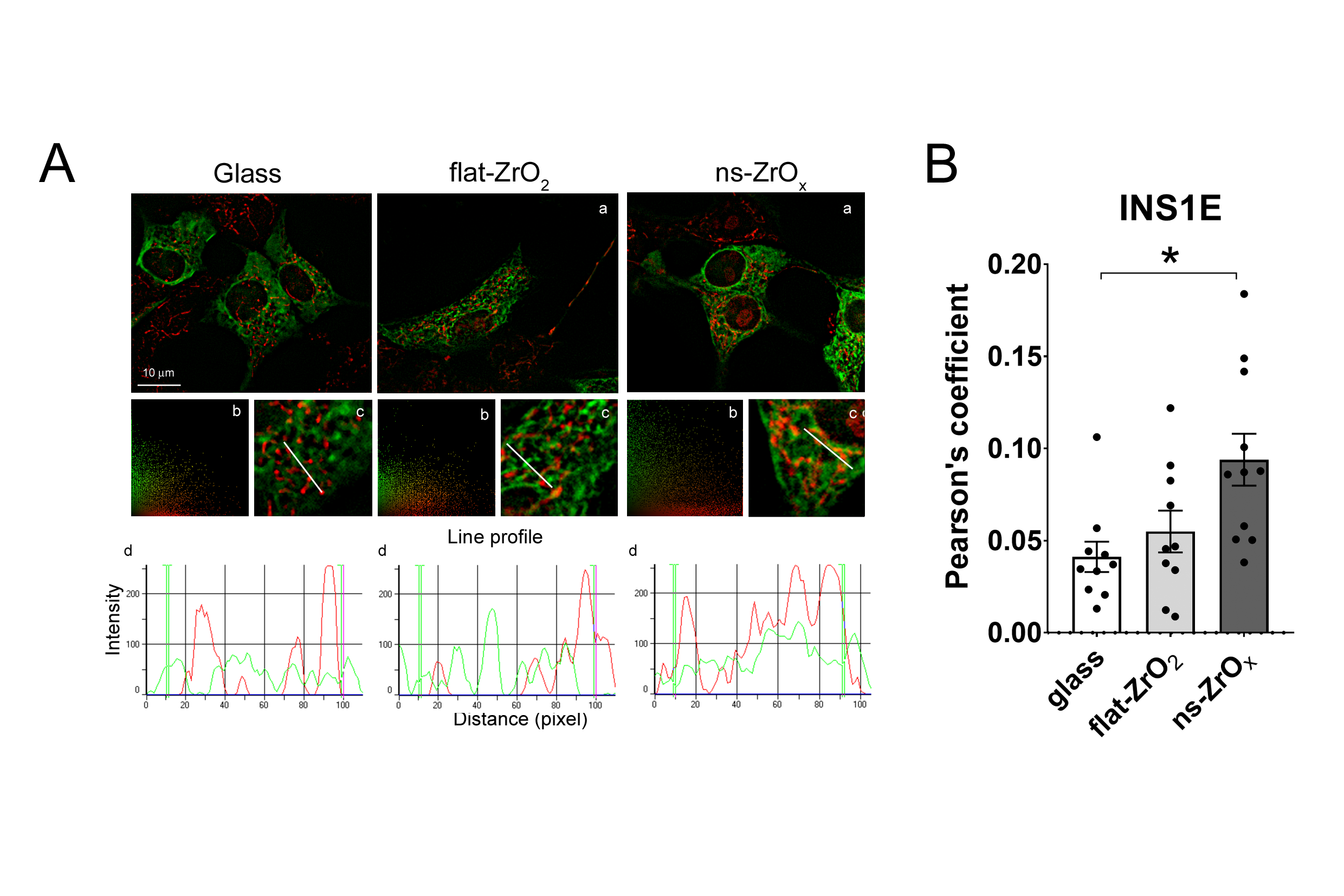

Supplement: FIGURE S3 — ER-Mitochondrial networks. INS1E plated on glass, flat-ZrO2 or ns-ZrOx substrates were transfected with ER-GFP (green) and after 48 h, the mitochondria were labeled with MitoSpyTM Orange (red). (A) (a) Representative images of ER-mitochondrial networks in cells grown on the indicated substrates. The yellow/orange staining highlights ER-mitochondria juxtaposition. Bar 10 μm. (b) Scatter plot analysis of image a. (c) Particular of panel a at higher magnification (3X). (d) plot of red and green fluorescence intensities along the line profile reported in c. (B) Bars report Pthe average Pearson’s colocalization coefficient value ± SE. Single values are reported. ∗p < 0.05 vs Glass. [file Image_3.tif]

Supplementary Blot 1

**A**

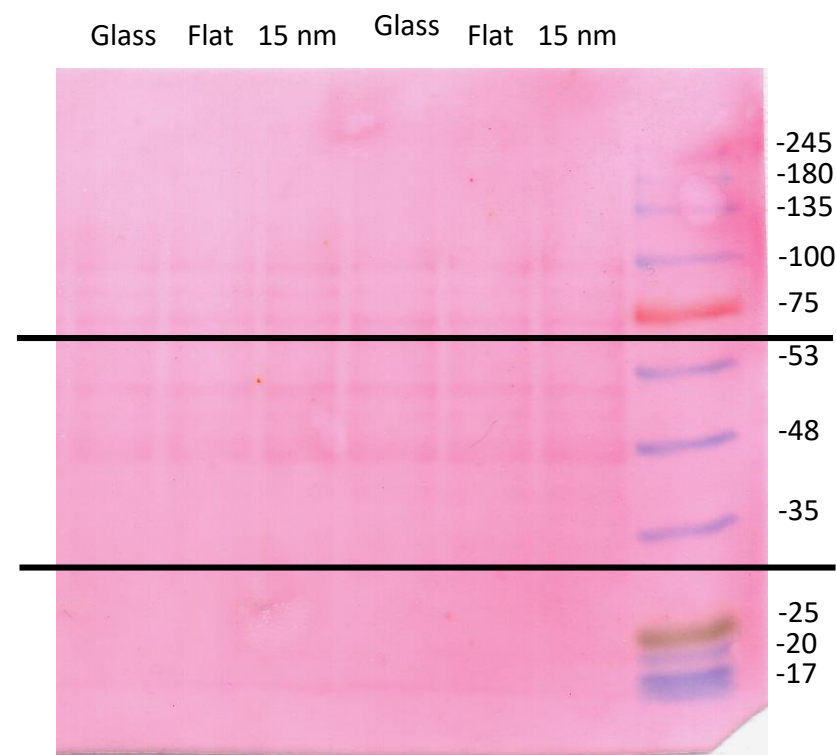

**B**

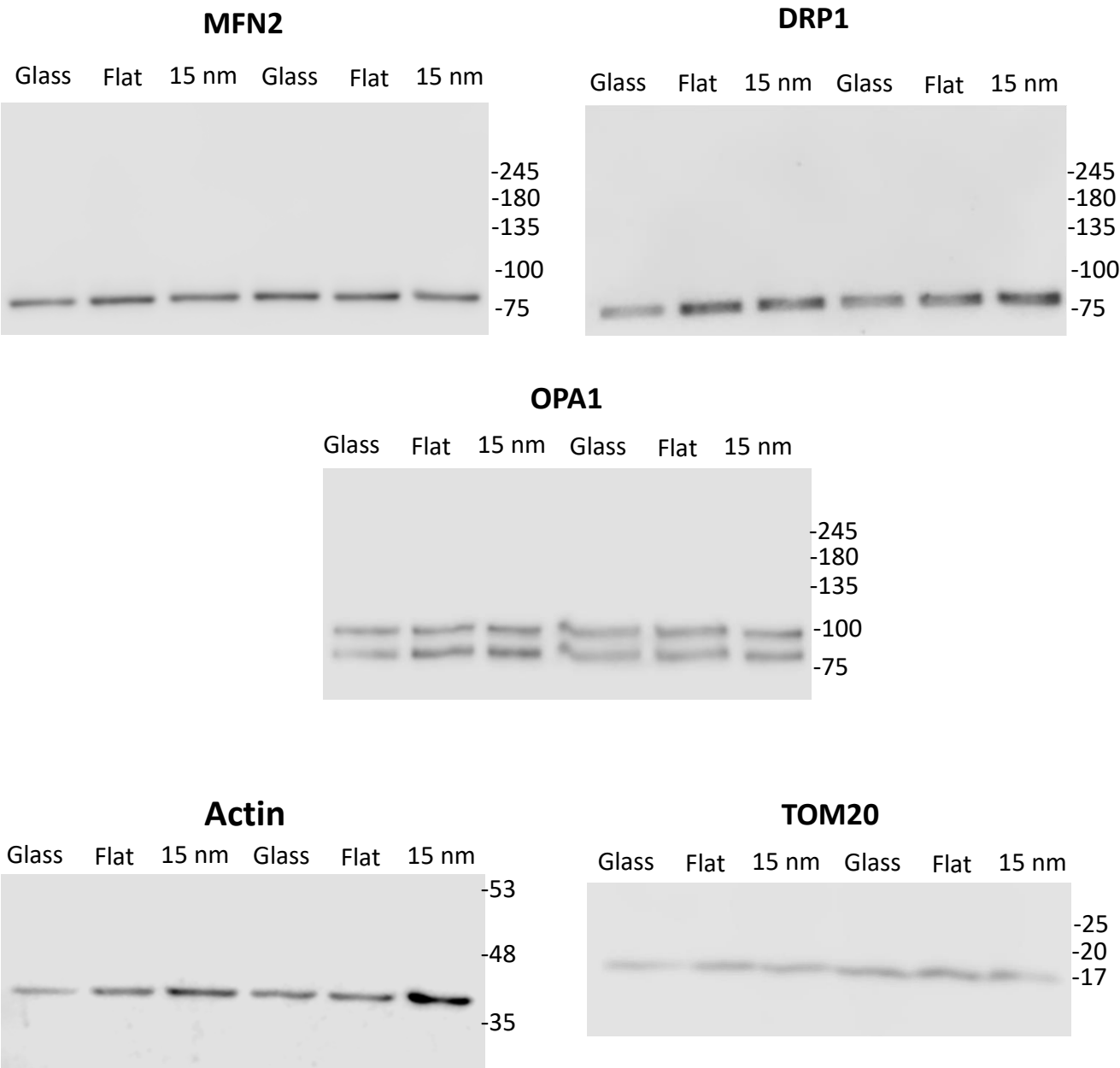

Supplementary Blot 2

**A**

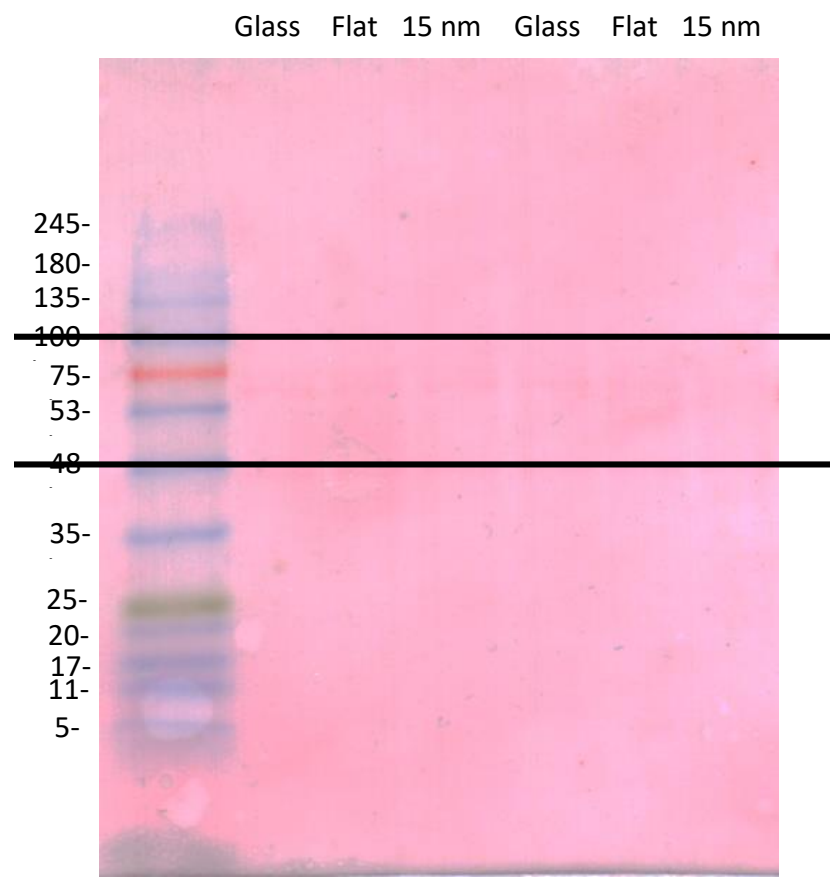

**B**

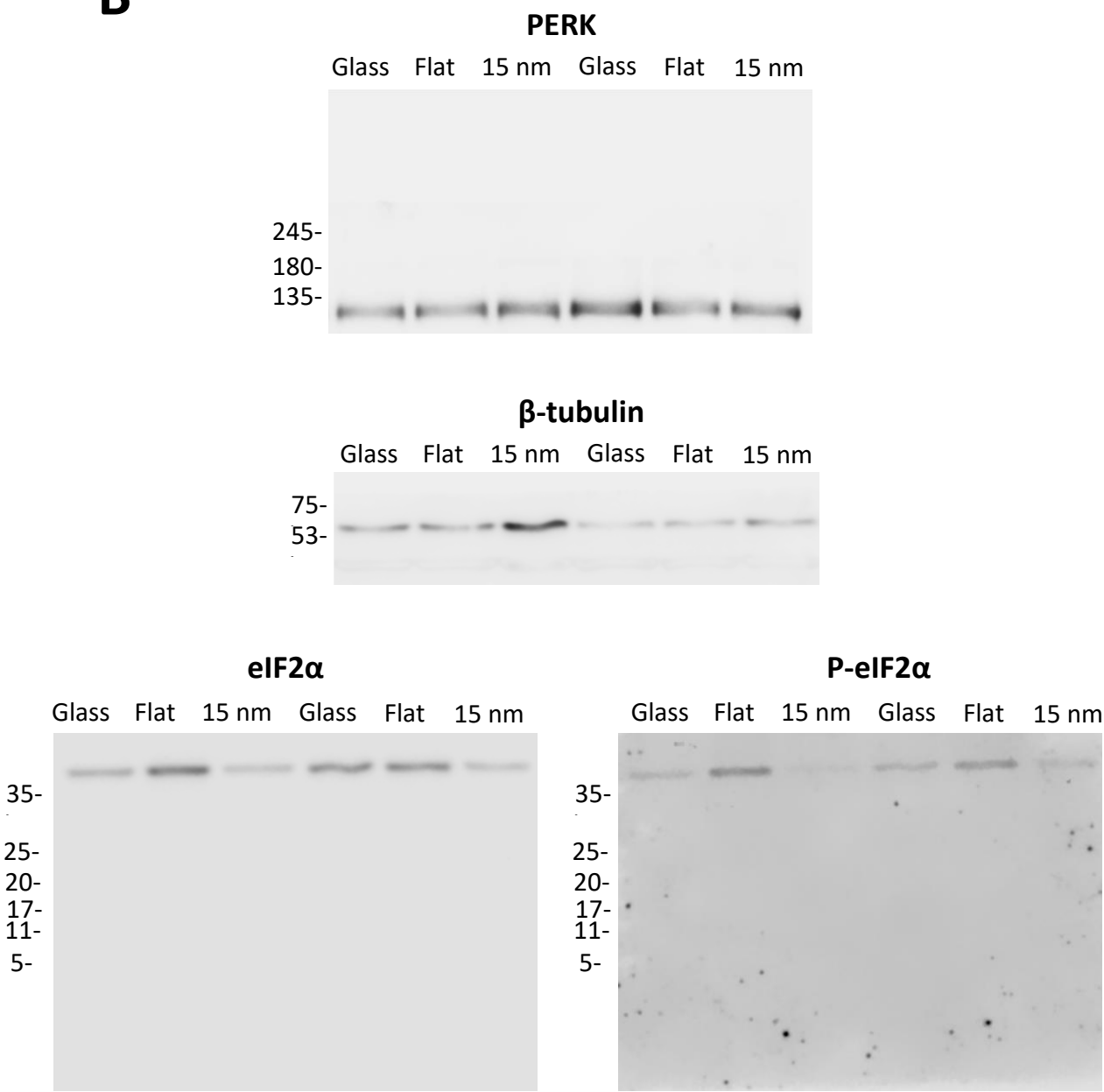

Supplementary Blot 3

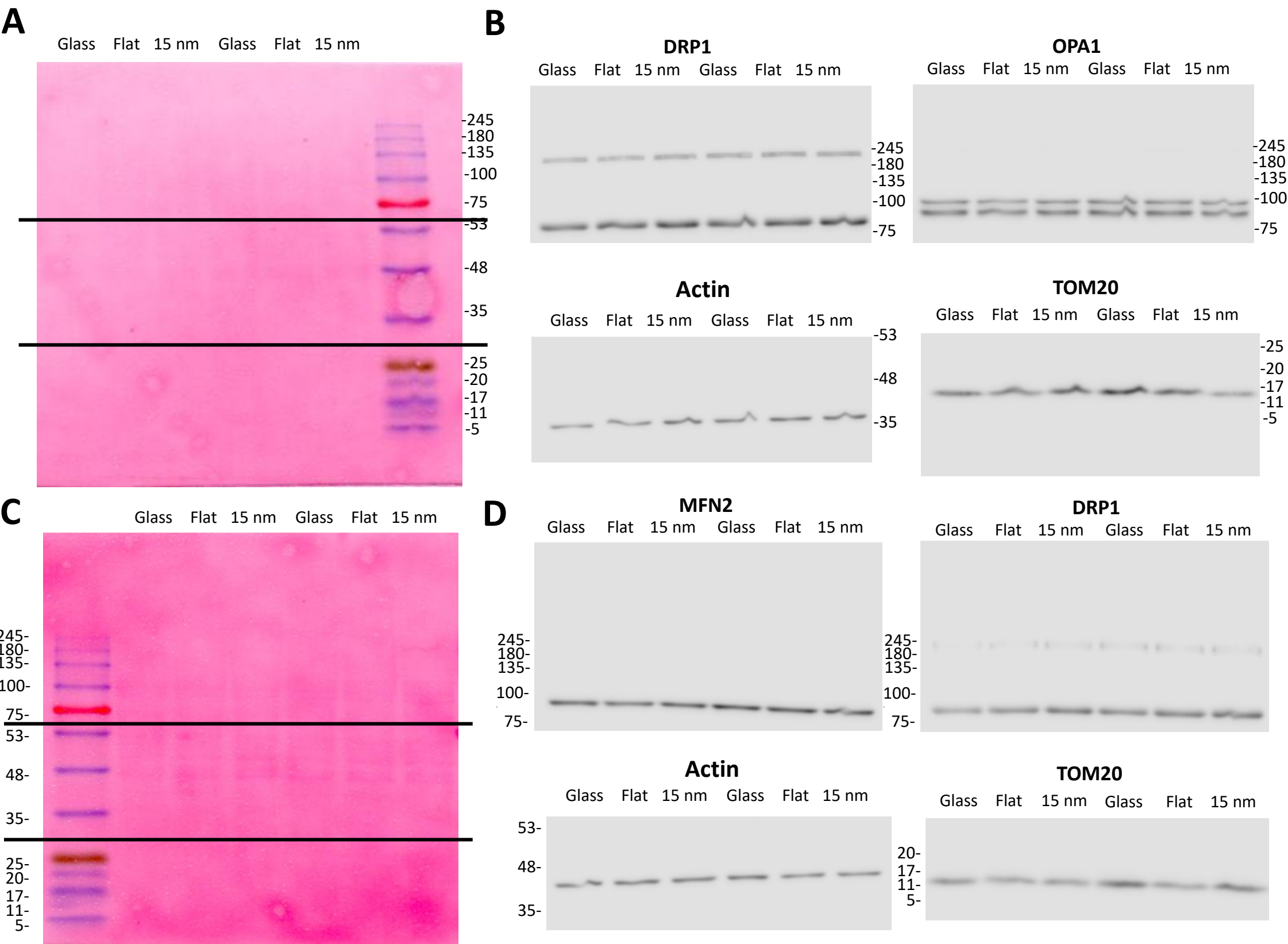

Supplement: BLOT S1 — βTC3 cells grown on the indicated substrates were lysed and 15 μg of proteins were resolved by 10% SDS-PAGE and transferred onto nitrocellulose membrane. (A) Representative image of ponceau staining; the molecular weight in kDa is reported on the right. (B) The membrane was divided into 3 parts: the upper part (>58 kDa) upper part was used to evaluate the expression of OPA1, MFN2, and DRP1—; the middle (30–58 kDa) and the lower (<30 kDa) parts to quantify actin and TOM20 expressions, respectively. [file Data_Sheet_1.pdf]
